# *Lycium barbarum* Glycopeptide Promotes Testosterone Synthesis and Glucose Metabolism in Leydig Cells of the Testis

**DOI:** 10.3390/biom15030425

**Published:** 2025-03-17

**Authors:** Jinlian Liang, Tianchan Peng, Jinrong Hu, Kwok Fai So, Hongyi Zhang, Guobing Chen, Yuan-Wei Zhang

**Affiliations:** 1 School of Life Sciences, Guangzhou University, Guangzhou 510006, China; liangjinl01@gzhu.edu.cn; 2 Department of Neurology, Affiliated Hospital of Jinan University, Guangzhou 510632, China; ptc1213@stu2022.jnu.edu.cn (T.P.); jinronghu1108@stu2023.jnu.edu.cn (J.H.); 3 Department of Microbiology and Immunology, Institute of Geriatric Immunology, School of Medicine, Jinan University, Guangzhou 510632, China; hongyizhang@jnu.edu.cn; 4 Institute of Clinical Research for Mental Health, The First Affiliated Hospital Jinan University, Guangzhou 510632, China; hrmaskf@hku.hk; 5 Center for Brain Science and Brain-Inspired Intelligence Guangdong-Hong Kong-Macao Greater Bay Area, Guangzhou 510515, China; 6 State Key Laboratory of Brain and Cognitive Science, Li Ka Shing Faculty of Medicine, the University of Hong Kong, Hong Kong SAR 999077, China

**Keywords:** *Lycium barbarum* glycopeptide, testosterone, Leydig cells, steroidogenic genes, proliferation

## Abstract

*Lycium barbarum* extracts have been shown to be effective in male reproductive protection and male infertility. However, its role in enhancing testicular function, such as testosterone synthesis, and the potential mechanism remain to be understood. To elucidate the effects of *Lycium barbarum* glycopeptide (LbGp) on testosterone synthesis, we isolated primary Leydig cells (LCs) from testes and performed RNA sequencing (RNA seq) on LCs treated with LbGp. In this study, we demonstrated that LbGp promoted testosterone synthesis in LCs both in vivo and in vitro. We also demonstrated that LbGp elevated adenosine 5′-triphosphate (ATP) synthesis and cell proliferation by enhancing glucose metabolism. Mechanistically, LbGp upregulated testosterone synthesis by suppressing TGF-β pathway and enhancing the expression of steroidogenic genes: *Cyp11a1*, *Hsd3b1*, *Hsd17b3*, *Star*, and *Sf-1*. These findings indicate that LbGp plays an important role in enhancing testicular function and promoting testosterone synthesis.

## 1. Introduction

Testosterone is the principal androgen in males, which is largely synthesized by Leydig cells (LCs) in the testes [1]. It plays a pivotal and indispensable role in triggering the initiation of spermatogenesis and ensuring its continuous and stable progression [2,3], promotes the development of male secondary sexual characteristics, and is closely related to male sexual desire [4]. A decline in serum testosterone levels may bring about testosterone deficiency (TD). As a well-recognized medical condition, TD has gained extensive attention [5]. It can affect multiple organ systems, thereby leading to significant health consequences and negative impacts on quality of life [6,7]. The occurrence of TD in men is related to aging. However, young men suffering from particular diseases will also be affected, including those with type 2 diabetes mellitus (T2DM), metabolic syndrome (MetS), obesity, cardiovascular disease (CVD), chronic obstructive pulmonary disease, kidney disease, and cancer [8,9,10]. Testosterone replacement therapy is the most direct and effective treatment for patients with TD. However, testosterone replacement therapy poses risks to the cardiovascular and cerebrovascular systems. Especially in men with a history of cardiovascular diseases or those at high risk, the incidence of major adverse cardiovascular events is higher with testosterone replacement therapy than with placebo. This highlights the recommendation that testosterone replacement therapy be used with caution [11,12,13,14]. In recent years, several studies have confirmed the potential of natural active ingredients to increase testosterone levels. *Lepidium meyenii*, *Trigonella foenum-graecum*, and *Tribulus arabica* have potential benefits for male fertility [15]. The extracts of these plants can help increase the levels of testosterone and luteinizing hormone in mice damaged by sodium glutamate, and promote male reproductive health and fertility [15]. In a clinical study, *Trigonella foenum-graecum* extract was proven to have a significant positive effect on the physiological aspects of sexual desire and may help maintain testosterone levels within the normal and healthy range [16]. Therefore, natural bioactive components that can increase testosterone levels are considered to be potential medical candidates for the treatment of TD.

*Lycium barbarum*, a traditional Chinese medicinal material, belongs to the *genus Lycium* of the *Solanaceae* family. In ancient Chinese medical classics such as Shen Nong’s Materia Medica from the Qin and Han dynasties (221 BC–220 AD), Thousand Ducat Prescriptions and Prescriptions from the Imperial Library from the Tang dynasty (618–907 AD), as well as the Compendium of Materia Medica written by Li Shizhen in the Ming Dynasty (1368–1644 AD), *Lycium barbarum* is listed as a medicine for treating diseases and promoting health. It was recorded for its therapeutic function of “strengthening the kidney”, “moisturizing the lungs”, “promoting male production”, and “brightening the eyes”. *Lycium barbarum* has the potential to enhance kidney function, promote respiratory system health, improve male fertility, and enhance eyesight according to the traditional Chinese medical system. There are also relevant reports on the use of *Lycium barbarum* in improving testicular function and treating male infertility. *Lycium barbarum* improved the spermatogenesis dysfunction caused by tripterygium glycosides by increasing the expression levels of CYP1A1, CYP17A1, AR, and SRD5A2 [17]. *Lycium barbarum* glycopeptide (LbGp) is a conjugated substance of polysaccharides and polypeptides extracted from *Lycium barbarum*, containing approximately 30% protein and monosaccharide components [18]. LbGp is regarded as a bioactive component with great significance. It was reported to exert multiple pharmacological actions, such as enhancing immunity [19] and antioxidation [20,21], alleviating anxiety [20], and demonstrating anti-cancer properties [22]. Recently, an increasing amount of evidence has indicated that the extracts of *Lycium barbarum* play a therapeutic role in the treatment of TD. *Lycium barbarum* polysaccharide (LBP), an extract of *Lycium barbarum*, has been shown to prevent a decline in serum testosterone levels and to improve erectile dysfunction in aged rats [23]. It also had a restorative effect on sexual dysfunction and reproductive damage in male mice with induced diabetes [24]. Studies show that di (2-ethylhexyl) phthalate (DEHP) caused damage to multiple organs and systems of organisms. LbGp, however, significantly improved the kidney and testicular damage caused by DEHP [25]. The underlying mechanism was proposed to be through regulation of the endocrine activity of the hypothalamic–pituitary–gonadal axis. However, so far, little information is available on the effect of LbGp on testosterone synthesis. We thus conducted a study to investigate the regulation of testosterone synthesis by LbGp.

## 2. Materials and Methods

### 2.1. Source of LbGp

The LbGp used in this study was purchased from Ningxia Tianren Goji Biotechnology (Zhongwei, China). LbGp is derived from the further extraction of *Lycium barbarum* polysaccharide (LBP). It has five main components, namely LbGp1-LbGp5 [18,21,25]. The provided LbGp was in powder form. We prepared it into a stock solution with a concentration of 2 mg/mL and used it after filtration.

### 2.2. Animals and Treatment

Male six-week-old C57BL/6J mice were procured from the Experimental Animal Center of Guangdong Province, China. The mice were housed in an environment maintained at a temperature of (25 ± 1) °C, with a relative humidity of 50–60% and a regular 12 h light/dark cycle. They had unrestricted access to the corresponding feed and drinking water. Animal experiments were carried out in full accordance with the National Institute of Health guidelines on the care and use of animals. Additionally, they were approved by the Institutional Animal Care and Use Committee of Jinan University (IACUC-20240403-22).

Twenty adult male C57BL/6J mice were randomly divided into two groups, with each group consisting of 10 mice: the sham group and the LbGp treatment group. LbGp mice were fed with LbGp (100 mg/kg per day) via intragastric administration for 12 weeks, and sham mice were given 0.01 M phosphate buffered saline (PBS).

### 2.3. Primary Leydig Cell Isolation

Primary Leydig cells (LCs) were obtained by isolating them from six-week-old male C57 mice [26]. Briefly, the mice were asphyxiated to death by introducing CO_2_. The testes were taken out and soaked in PBS. After the tunica albuginea was removed, the testes were placed in DMEM medium (KeyGen, Nanjing, China, KGL1206−500) containing 1 mg/mL collagenase VI and immediately digested in a shaking water bath at 37 °C for 10 min. Then, DMEM medium supplemented with 10% fetal bovine serum (FBS) (KeyGen, Nanjing, China, KGL3002−50) was used to stop digestion. The cell supernatant was passed through a 40 μm cell strainer for filtration and subsequently subjected to centrifugation at 200× *g* for 3 min. The cells were re-suspended in fresh culture medium and then incubated at 37 °C with 5% CO_2_ for 1 h. Subsequently, the culture medium was replaced to get rid of non-adherent cells, after which the cells were cultured for another 48 h.

### 2.4. Western Blots

Leydig cells and testis tissues were lysed in RIPA solution (Fude, Hangzhou, China, FD008) containing 1 mM PMSF and protease inhibitor cocktail (Yeasen, Shanghai, China, 20124ES03) on ice for 30 min. The lysates underwent centrifugation at a temperature of 4 °C, with a centrifugal force set at 12,000× *g* for a period of 15 min. Electrophoresis of 30 μg of proteins was performed on a 10% SDS-PAGE gel, followed by transferring them onto PVDF membranes. Antibodies (LHCGR, SF-1, CYP11A1, CYP17A1, HSD3B1, HSD17B3, and StAR) diluted at a ratio of 1:1000 were added to the membranes and incubated for 12 h at 4 °C. The membrane was washed 5 times with tris buffered saline with Tween 20 (TBST), with each wash lasting 7 min. Then, it was incubated with the secondary antibody for one hour and subsequently washed another 5 times. Finally, the membrane was incubated with enhanced chemiluminescence (ECL) solution (Yeasen, Shanghai, China, 36222ES60) to detect the protein levels. Information on the antibodies is written in Table S1.

### 2.5. Testosterone Measurements

The testosterone level in the culture supernatant of LCs was detected by using an iodine [^125^I] testosterone radioimmunoassay kit (Beijing North Institute of Biotechnology, Beijing, China, S10940093). Briefly, 50 μL of cell supernatant sample were added to a 5 mL tube containing 100 μL of ^125^I-labeled testosterone derivative and 100 μL of testosterone antibody. After being thoroughly mixed, the solution was incubated in a 37 °C water bath for 1 h. After that, the addition of 500 μL of an immunological separating agent was carried out, followed by incubation at room temperature for 15 min. The tubes were centrifuged at 3500× *g* for 15 min. Following the discarding of the supernatant, the radioactive counts of each precipitation tube were determined with a γ counter.

### 2.6. Quantitative Real-Time Polymerase Chain Reaction (qRT-PCR)

Total RNA was isolated from Leydig cells through the utilization of Trizol solution (Thermo Fisher, Waltham, MA, USA, 15596026CN). Complementary DNA (cDNA) was obtained from 1 μg of total RNA through reverse transcription using an RNA reverse-transcription kit (Vazyme, Nanjing, China, R333−01). Two μL of the diluted cDNA were used as the template. It was placed in a total reaction volume of 20 μL, which consisted of 10 μL of 2 × chamQ SYBR qPCR master mix (Vazyme, Nanjing, China, Q311−02), 0.4 μL each of forward and reverse primers, and 7.2 μL of nuclease-free water. After that, the qRT-PCR procedure was carried out. The initial activation step was conducted at 95 °C for 30 s. This was then followed by 40 cycles, with each cycle consisting of a 10 s denaturation step at 95 °C and a 30 s annealing/extension step at 60 °C. The relative gene expressions were calculated by the 2^−ΔΔCt^ method after normalization to β-actin. The primer sequences are written in Table S2.

### 2.7. Immunofluorescence

Leydig cells underwent a 48 h treatment with LbGp. Post-treatment, the cells were fixed in 4% paraformaldehyde for 10 min and then subjected to three rounds of PBS washes. To facilitate better antibody penetration, the cells were treated with 0.5% Triton X-100 for 10 min. Subsequently, non-specific binding was minimized through a 1 h blocking step at room temperature using 5% bovine serum albumin (BSA). The cells were then incubated overnight at 4 °C with primary antibodies (Table S1) diluted to a ratio of 1:200. After this incubation, they were rinsed three times with PBS. After the primary antibody step, a 1 h incubation at room temperature with the secondary antibody (Abcam, Cambridge, UK, Ab150077) was carried out. The cells were washed three additional times with PBS. Ultimately, the stained cells were subjected to an imaging process. A confocal microscope (Nikon, Tokyo, Japan, NIS−Elements AR) was utilized to capture images of these stained cells, enabling further observation and analysis.

### 2.8. Adenosine 5′-Triphosphate (ATP) Measurement

The ATP levels of the cells were detected using an ATP assay kit (Beyotime, Shanghai, China, S0026). Leydig cells were treated with 0, 50, 100, 200, 300, and 400 μg/mL LbGp for 48 h. After being collected in a 1.5 mL centrifuge tube, the cells were thoroughly lysed by the addition of 200 μL of lysis buffer. Following this step, centrifugation of the sample was carried out at 12,000 rpm and 4 °C for 5 min. Then, 20 μL of cell supernatant were taken and added to the ATP detecting solution. The luminescence values were detected by a multifunctional microplate reader.

### 2.9. Cell Proliferation Assays

For the purpose of conducting an in vitro assessment of cell viability, the Cell Counting Kit-8 (CCK8, Beyotime, Shanghai, China, C0039) was applied to determine the viability status of the cells. After being plated into 96-well plates at a seeding density of 2000 cells per well, Leydig cells were incubated for 24 h. Then, the cells were treated with 0, 50, 100, 200, 300, and 400 μg/mL of LbGp for 72 h. The CCK8 reagent was added to the cells and they were incubated at 37 °C for 2 h. Finally, the OD values were measured at a wavelength of 450 nm.

### 2.10. EdU Incorporation Assay

The proliferation of Leydig cells was detected with an EdU incorporation assay using an EdU cell proliferation kit (KeyGen, Nanjing, China, KGA9602−100). At a seeding density of 5 × 10^4^ cells per well, Leydig cells were seeded onto 24-well plates. After the cells had adhered to the surface, they were treated with 0, 50, 100, and 300 μg/mL LbGp for 24 h. Then, replaced the original culture medium with complete DMEM culture medium containing 10 mM EdU, and continued culturing for 24 h. As the cells divided, EdU was incorporated into the nucleus. Cells that were positive for EdU were newly generated cells. The cells were fixed and stained following the instructions of the manufacturer, and finally imaged with a confocal microscope (Nikon, Tokyo, Japan, NIS−Elements AR) and analyzed with Image J software (1.48v/Java 1.6.0 20).

### 2.11. Statistical Analysis

The data, which were derived from at least three independent experiments, are presented in the form of the mean along with the standard error of the mean (SEM). In GraphPad Prism 8, the one-way ANOVA or *t*-test function was employed to assess the differences in means. Statistical significance was determined based on the *p*-values: differences between the means were regarded as statistically significant when * *p* < 0.05, ** *p* < 0.01, or *** *p* < 0.001.

## 3. Results

### 3.1. LbGp Enhances Leydig Cell Proliferation and Testosterone Synthesis

**Figure 1:**
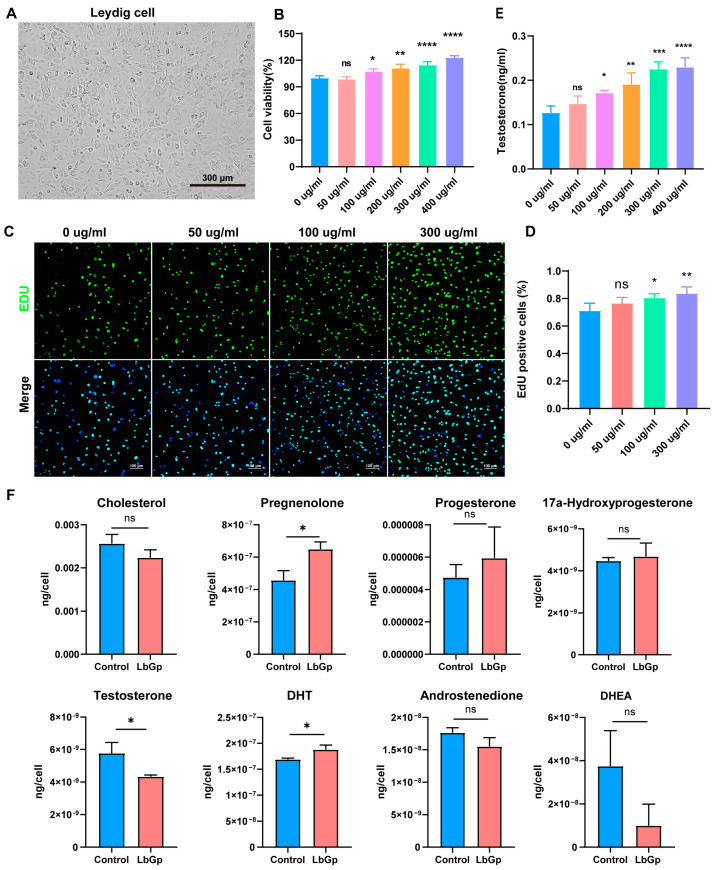
LbGp enhances Leydig cell proliferation and testosterone synthesis. (**A**) The primary LCs isolated from 6-week-old C57 mice, scale bar, 300 μm. (**B**) Cell viability of LCs treated with 0, 50, 100, 200, 300, and 400 μg/mL LbGp for 72 h, detected by CCK8. (**C**) The proliferation of LCs treated with 0, 50, 100, and 300 μg/mL LbGp for 72 h, detected by EdU assay. (**D**) The statistical results of EdU-positive cells in Figure (**C**). (**E**) The testosterone concentrations in supernatant of LCs treated with 0, 50, 100, 200, 300, and 400 μg/mL LbGp for 48 h, detected by radioimmunoassay. (**F**) The concentrations of various steroids in LCs treated with or without 100 μg/mL LbGp for 48 h, detected by UHPLC-MS/MS. The data are shown as the mean ± SEM and which are derived from at least three independent experiments. * *p* < 0.05, ** *p* < 0.01, *** *p* < 0.001, **** *p* < 0.0001, “ns” means not significant.

Testosterone, a hormone of utmost significance in the male reproductive system, is mainly synthesized by Leydig cells (LCs) in the testes. Here, we isolated primary LCs (Figure 1A) from six-week-old C57 mice testes to study the effect of LbGp on male reproduction. In order to examine the influence of LbGp on LC proliferation, we treated LCs with 0, 50, 100, 200, 300, and 400 μg/mL of LbGp for 72 h and then conducted a CCK8 assay. The results show that LbGp enhanced the viability of LCs (Figure 1B). Moreover, the EdU incorporation assay demonstrated that LbGp at either 100 μg/mL or 300 μg/mL concentration significantly promoted the proliferation of LCs (Figure 1C,D). Additionally, we measured the testosterone contents in the supernatants of LCs treated with LbGp for 48 h by radioimmunoassay and found that LbGp elevated testosterone secretion by LCs (Figure 1E). Testosterone is a steroid androgen produced by the step-by-step catalysis of cholesterol by a series of steroid-synthesizing enzymes. To fully investigate how LbGp regulates the synthesis of testosterone, we conducted targeted steroid detection on LCs treated with 100 μg/mL LbGp via an ultra-high-performance liquid chromatography coupled with tandem mass spectrometry (UHPLC-MS/MS). With a total 38 steroids, 25 steroids were successfully detected (Figure 1F and Figure S1A and Table S3). The results show that LbGp up-regulated the synthesis of pregnenolone, an intermediate in testosterone synthesis, and elevated the conversion of dihydrotestosterone (DHT), an activated form of testosterone. Meanwhile, we also observed a decrease in intracellular testosterone, which could be attributed to the conversion of testosterone into dihydrotestosterone and the secretion of a large portion of testosterone into the cell supernatant (Figure 1E). These results show that LbGp promoted the proliferation of LCs and testosterone synthesis.

### 3.2. LbGp Up-Regulates the Expression of Steroidogenic Genes

**Figure 2:**
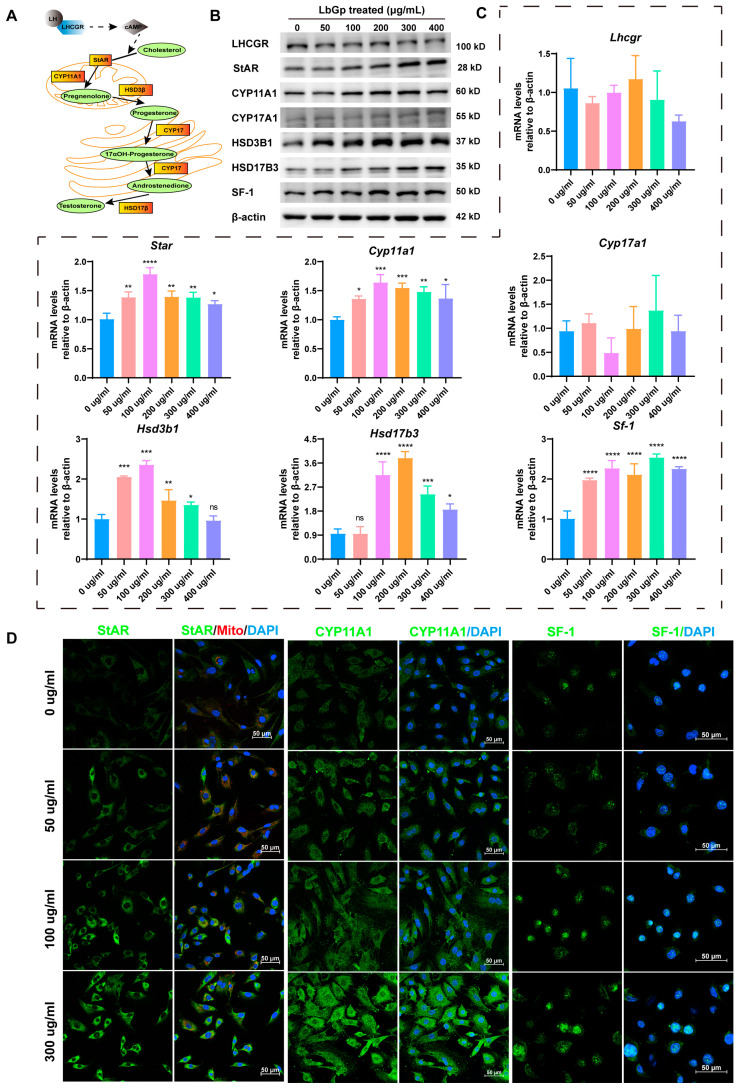
LbGp increases the expression of steroidogenic genes in LCs. (**A**) Schematic diagram of the testosterone synthesis. (**B**) The protein levels of LHCGR, StAR, CYP11A1, CYP17A1, HSD3B1, HSD17B3, and SF-1 in LCs treated with LbGp, detected by Western blotting. (**C**) The mRNA levels of *Lhcgr*, *Star*, *Cyp11a1*, *CYP17a1*, *Hsd3b1*, *Hsd17b3*, and *Sf-1* in LCs treated with LbGp, detected by qRT-PCR. (**D**) Detection of StAR, CYP11A1, and SF-1 in LCs treated with 0, 50, 100, and 300 μg/mL LbGp for 48 h by immunofluorescence. The red signals are mitochondrion. The data are shown as the mean ± SEM and are derived from at least three independent experiments. * *p* < 0.05, ** *p* < 0.01, *** *p* < 0.001, **** *p* < 0.0001, “ns” means not significant. Western blot original images can be found in Supplementary File S1.

Testosterone synthesis is a complex process. It involves the activation of the cAMP and cholesterol transportation to the inner mitochondrial membrane. Subsequently, it undergoes successive conversions in mitochondria and the endoplasmic reticulum (Figure 2A). To delve deeper into the mechanism whereby LbGp controls the synthesis of testosterone in LCs, we treated LCs with various concentrations of LbGp and detected the proteins and genes related to testosterone synthesis. The results show that LbGp significantly up-regulated the protein and mRNA levels of steroidogenic acute regulatory (StAR), cytochrome P450 family 11 subfamily A member 1 (CYP11A1), hydroxy-delta-5-steroid dehydrogenase, 3β-and steroid delta-isomerase 1 (HSD3B1), hydroxysteroid 17β dehydrogenase 3 (HSD17B3), and steroidogenic factor 1 (SF-1), but did not alter the expression of the luteinizing hormone receptor (LHCGR) or cytochrome P450 family 17 subfamily A member 1 (CYP17A1) (Figure 2B,C and Figure S2A). In addition, the results of immunofluorescence revealed that LbGp dramatically elevated the protein level of StAR, CYP11A1, and SF-1 in LCs (Figure 2D). StAR assumes a crucial position in the process of steroidogenesis. Its principal function is to mediate the translocation of cholesterol from the outer to the inner mitochondrial membrane. This translocation event represents the rate-limiting step in the biosynthesis of steroid hormones, thereby exerting a significant influence on the overall efficiency of steroidogenesis [27]. CYP11A1, CYP17A1, HSD3B1, and HSD17B3 are enzymes that catalyze testosterone synthesis. However, SF-1 regulates the levels of key enzymes implicated in testosterone synthesis, including CYP11A1, CYP17A1, HSD3B1, and HSD17B3 [28]. SF-1 activates their transcription, leading to the production of the corresponding enzymes. The above experimental results indicate that LbGp promoted the transport of cholesterol into mitochondria by increasing the expression of StAR. Additionally, it also increased the expression of steroidogenic proteins (CYP11A1, HSD3B1, HSD17B3, and SF-1), thereby increasing the synthesis of testosterone.

### 3.3. Profiling Gene Expression of Leydig Cells Treated with LbGp

**Figure 3:**
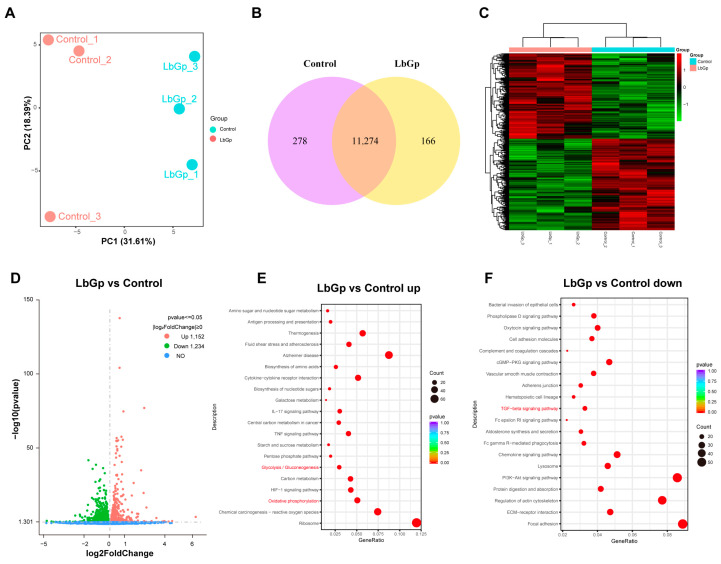
Gene expression profile of Leydig cells treated with LbGp. (**A**) Principal component analysis (PCA) visualization of the transcriptional variations in LCs treated with LbGp. (**B**) Venn diagram showing the number of genes between two groups (control and LbGp groups). (**C**) Heat map representing the expression of the 2386 DEGs. (**D**) A volcano plot of genes in the two groups. Genes that are up-regulated, down-regulated, and with no significant difference are represented by red, green, and blue dots, respectively. (**E**) KEGG pathway enrichment for genes up-regulated in LbGp-treated LCs. The dot size represents the number of DEGs enriched in the corresponding pathway. (**F**). KEGG pathway enrichment for genes down-regulated in LbGp-treated LCs. The dot size represents the number of DEGs enriched in the corresponding pathway.

For the purpose of delving into the mechanism whereby LbGp modulates testosterone synthesis and LC proliferation, we conducted RNA transcriptome sequencing on LCs treated with or without 100 μg/mL of LbGp for 48 h. We performed principal component analysis (PCA) to visualize the transcriptional changes among the samples. We found that all the biological replicates of LCs treated with LbGp clustered together within a 95% confidence interval and were separated from the untreated ones (Figure 3A), indicating the distinct transcriptional patterns of the cells treated with LbGp. The Venn diagram (Figure 3B) showed that there were 11,274 common genes between the two groups, with 278 and 166 genes being unique to the control and the LbGp group, respectively. From these common genes, 2386 differentially expressed genes (DEGs) were confirmed. In comparison with the control group, the expression levels of 1152 genes were increased and 1234 genes were decreased (Figure 3C,D). KEGG analyses revealed that LbGp treatment activated the glycolysis and the oxidative phosphorylation pathway (Figure 3E), while suppressing the transforming growth factor-β (TGF-β) signaling pathway (Figure 3F). Glycolysis and oxidative phosphorylation are the main pathways of glucose metabolism. The enhancement in glucose metabolism is conducive to obtaining more energy to promote cell proliferation. In addition, TGF-β, which is part of the TGF-β signaling pathway, plays a crucial role in testosterone synthesis. It influences the expression of proteins relevant to testosterone synthesis by modulating SF-1.

### 3.4. LbGp Promotes Glucose Metabolism and ATP Synthesis in Leydig Cells

**Figure 4:**
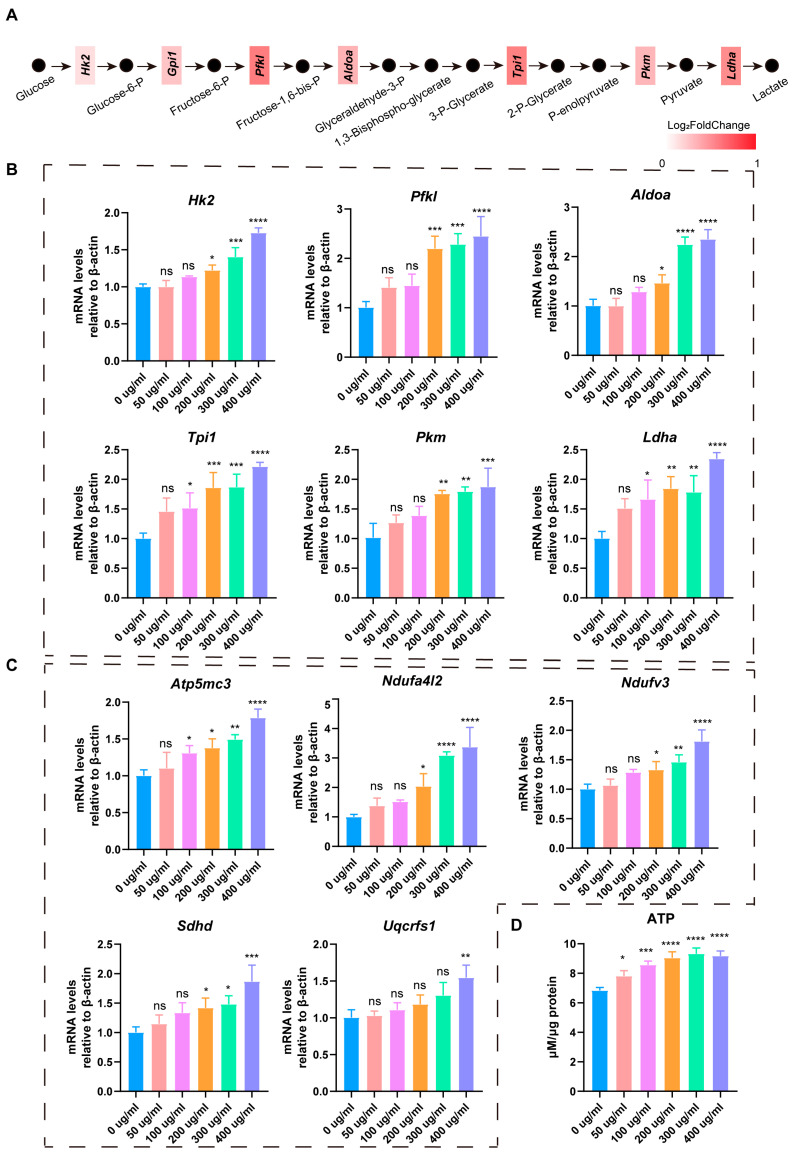
LbGp promotes glucose metabolism and ATP synthesis in Leydig cells. (**A**) Changes in the genes of the glycolytic pathway between LCs treated with LbGp and the control cells. (**B**) The expression of genes associated with glycolysis in LCs treated with 0, 50, 100, 200, 300, and 400 μg/mL LbGp for 24 h, tested by qRT-PCR. (**C**) The expression of genes involved in oxidative phosphorylation pathway in LCs treated with 0, 50, 100, 200, 300, and 400 μg/mL LbGp for 24 h, measured by qRT-PCR. (**D**) The ATP levels of LCs treated with 0, 50, 100, 200, 300, and 400 μg/mL LbGp for 48 h. The data are shown as the mean ± SEM and are derived from at least three independent experiments. * *p* < 0.05, ** *p* < 0.01, *** *p* < 0.001, **** *p* < 0.0001, “ns” means not significant.

In the process of gene expression profiling analysis, we found that LbGp activated the glycolysis and oxidative phosphorylation processes in LCs (Figure 3E and Figure 4A). We treated LCs with 0, 50, 100, 200, 300, and 400 μg/mL LbGp for 24 h and detected the mRNA level of genes involved in glycolysis and oxidative phosphorylation by qRT-PCR. The data revealed that the mRNA level of genes that are involved in the glycolysis, such as hexokinase 2 (*Hk2*), phosphofructokinase liver type (*Pfkl*), aldolase, fructose-bisphosphate A (*Aldoa*), triosephosphate isomerase 1 (*Tpi1*), pyruvate kinase M1/2, and lactate dehydrogenase A (*Ldha*), was upregulated (Figure 4B). The expression of genes related to oxidative phosphorylation, such as ATP synthase membrane subunit c locus 3 (*Atp5mc3*), NADH dehydrogenase (ubiquinone) 1 alpha subcomplex, 4-like 2 (*Ndufa4l2*), NADH: ubiquinone oxidoreductase subunit V3 (*Ndufv3*), succinate dehydrogenase complex subunit d (*Sdhd*) and ubiquinol-cytochrome c reductase, and Rieske iron-sulfur polypeptide 1 (*Uqcrfs1*), was also increased (Figure 4C). These findings indicate that LbGp promoted glucose metabolism in LCs by activating the glycolysis and oxidative phosphorylation processes. Furthermore, we collected LCs treated with 0, 50, 100, 200, 300, and 400 μg/mL LbGp for 48 h and detected the intracellular ATP contents. The results demonstrate that LbGp enhanced the synthesis of ATP in LCs (Figure 4D). Glucose metabolism is a major pathway for energy production in cells. When glucose metabolism is increased, cells decompose more glucose, releasing a large amount of ATP. This additional energy can support various cellular activities, including cell division, movement, and biosynthesis. Therefore, our results revealed that LbGp promoted LC proliferation by enhancing cellular glucose metabolism and generating more energy.

### 3.5. LbGp Promotes the Expression of Steroidogenic Genes by Inhibiting the TGF-β Signaling Pathway

**Figure 5:**
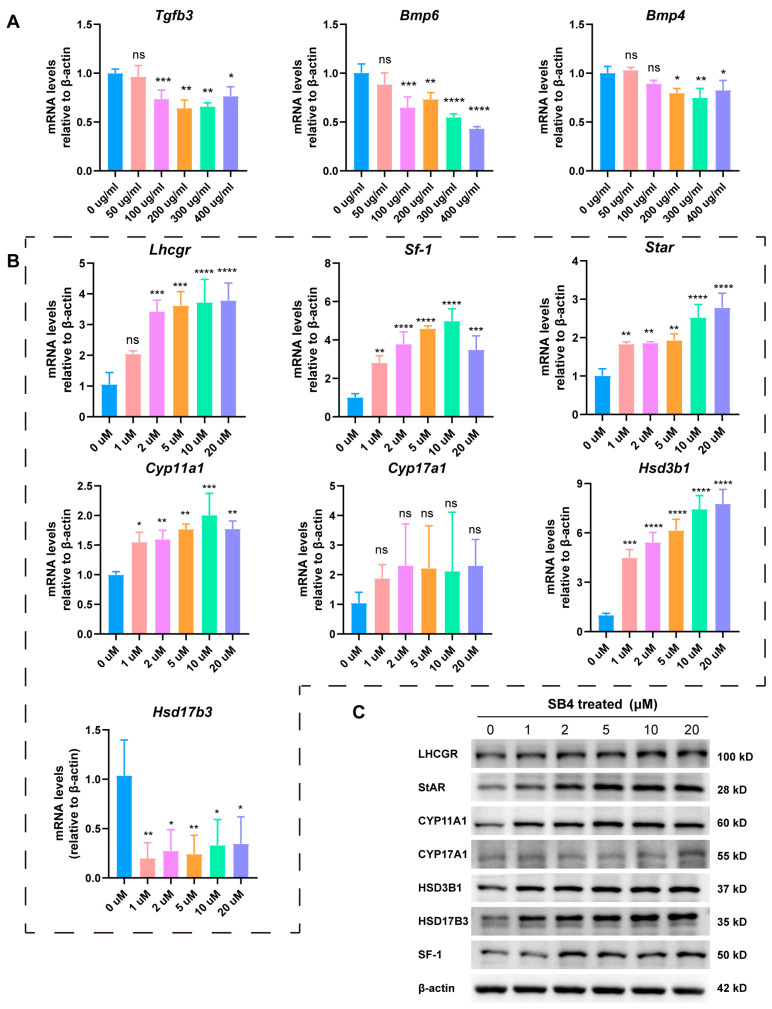
LbGp suppresses the TGF-β signaling pathway in Leydig cells. (**A**) The expression of genes involved in TGF-β pathway in LCs treated with 0, 50, 100, 200, 300, and 400 μg/mL LbGp for 24 h, tested by qRT-PCR. (**B**) The mRNA levels of steroidogenic genes in LCs treated with 0, 1, 2, 5, 10, and 20 μM SB4 for 24 h, detected by qRT-PCR. (**C**) The expression of steroidogenic proteins in LCs treated with 0, 1, 2, 5, 10, and 20 μM SB4 for 48 h, detected by Western blotting. The data are shown as the mean ± SEM and are derived from at least three independent experiments. * *p* < 0.05, ** *p* < 0.01, *** *p* < 0.001, **** *p* < 0.0001, “ns” means not significant. Western blot original images can be found in Supplementary File S1.

In LCs treated with LbGp, the TGF-β signaling pathway was suppressed (Figure 3E). It has been verified that the TGF-β signaling pathway exerts a down-regulating effect on testosterone synthesis. The TGF-β1/ALK5/Smad3 pathway inhibited the mRNA level of steroidogenic genes in testicular LCs by inhibiting the transcriptional activation of Nur77 [29]. It was identified that TGF-β reduced the transcription level of steroidogenic factor 1 (SF-1) in Y-1 adrenocortical cells [30]. TGF-β1 exerts its regulatory function through Smad2. Specifically, by up-regulating particular microRNAs, it suppresses the expression of SF-1 and LRH-1 that occurs at the post-transcriptional stage [31]. We examined the TGF-β signaling pathway by qRT-PCR and found that LbGp decreased the expression of *Tgfb3*, *Bmp4*, and *Bmp6* in LCs, but did not change the mRNA level of Tgfb2 (Figure 5A and Figure S3A). Furthermore, we treated LCs with the TGF-β receptor inhibitor SB431542 (SB4) and analyzed the expression of the steroidogenic genes using qRT-PCR and Western blotting. Our research uncovered that suppressing the TGF-β signaling pathway increased the expression of LHCGR, CYP11A1, HSD3B1, HSD17B3, and SF-1 (Figure 5B,C and Figure S3B). Consistent with the results obtained after treating the cells with LbGp, these results demonstrate that LbGp promoted the expression of steroidogenic genes by suppressing the TGF-β signaling pathway, consequently enhancing the synthesis of testosterone.

### 3.6. LbGp Increases the Expression of Steroidogenic Genes In Vivo

**Figure 6:**
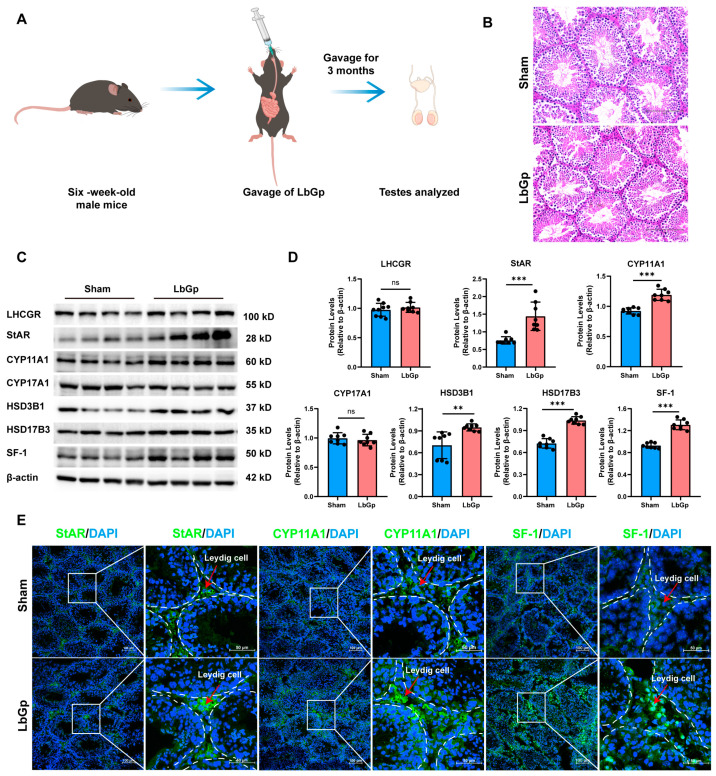
LbGp promotes the expression of steroidogenic proteins in the testes. (**A**) Schematic diagram of intragastric administration of LbGp to C57 mice. (**B**) HE staining of testes of the sham and LbGp-treated mice. (**C**) The expression of steroidogenic proteins in the testes of the sham and LbGp-treated mice, detected by Western blotting. (**D**) The statistical analysis of the protein level of steroidogenic proteins in Figure (**C**). (**E**) Detection of StAR, CYP11A1, and SF-1 in the testes of the sham and LbGp-treated mice by immunofluorescence. The data are shown as the mean ± SEM and are derived from at least three independent experiments. ** *p* < 0.01, *** *p* < 0.001, “ns” means not significant. Western blot original images can be found in Supplementary File S1.

To investigate the effects of LbGp on male reproduction, six-week-old C57 mice were administered by gavage with LbGp at a dose of 100 mg/kg per day, while sham mice were fed PBS. The gavage treatment with LbGp/PBS lasted for 3 months (Figure 6A). The testes of mice were then collected for further analysis. Hematoxylin–eosin (HE) staining was carried out on testis tissue samples. The results indicate that there was no inflammation or damage in the testes (Figure 6B), suggesting that LbGp had no reproductive toxicity. Compared with the sham group, the seminiferous tubules in the LbGp treatment group were denser, and the arrangement of the spermatogenic cell layer was also more compact, indicating that the number of cells may have increased (Figure 6B). Western blot analysis demonstrated that the protein levels of steroidogenic proteins, namely StAR, CYP11A1, HSD3B1, HSD17B3, and SF-1, in the LbGp-treated mice were remarkably higher compared to those in the sham mice (Figure 6C,D). However, the protein levels of LHCGR and CYP17A1 had little change (Figure 6C,D). Immunofluorescence analysis also showed that LbGp significantly elevated the protein levels of StAR, CYP11A1, and SF-1 in the interstitium of the testes (Figure 6E). These results are consistent with those obtained when LbGp was used to treat LCs in vitro. When all these results are considered together, it is clearly established that LbGp played a role in modulating testosterone synthesis. It achieved this by promoting the expression of key steroidogenic proteins: StAR, CYP11A1, HSD3B1, HSD17B, and SF-1, which are crucial for the normal process of testosterone synthesis.

## 4. Discussion

LbGp from *Lycium barbarum* is regarded as an active ingredient with great medicinal value. In this research, we obtained LCs from mice testes and treated them with LbGp. We found that LbGp promoted the secretion of testosterone and cell proliferation. Furthermore, we discovered that LbGp increased the intracellular levels of pregnenolone, an intermediate product in the testosterone synthesis process, and dihydrotestosterone. Our study demonstrated that LbGp promoted testosterone synthesis, indicating that LbGp is a promising medical candidate for treating testosterone deficiency.

Multiple studies have reported that *Lycium barbarum* and its extracts carry out multiple pharmacological actions on male reproduction. *Lycium barbarum* caused an up-regulation in the expression of CYP19A1, CYP17A1, AR, and SRD5A2, which in turn increased the level of serum testosterone to combat infertility [17]. In rats treated with doxorubicin, LBP was effective in decreasing the oxidative stress within the testes and safeguarded the testes against their specific toxicity [32]. Polysaccharides isolated from *Lycium barbarum* alleviated diabetic testicular dysfunction by decreasing abnormal autophagy via the PI3K/Akt signal in male mice [33]. In 18-month-old rats, the extract of *Lycium barbarum* salvaged erectile dysfunction by increasing endothelial and neuronal nitric oxide synthases, improving oxidative stress, and elevating the level of serum testosterone [23]. The vast majority of these studies investigated the impact of the *Lycium barbarum* extract on testosterone synthesis at the animal level. Leydig cells are the main producers of testosterone. We isolated Leydig cells from the testes of mice and firstly demonstrated that LbGp can directly act on Leydig cells to promote testosterone synthesis.

In this article, we demonstrated that LbGp directly promotes the transcription of steroidogenic genes *Cyp11a1*, *Hsd3b1*, *Hsd17b3*, *Star*, and *Sf-1* by suppressing TGF-β pathway, thereby facilitating the synthesis of testosterone. The TGF-β pathway is involved in regulating steroid synthesis in both female and male animals. In female reproduction, the knockdown of SMAD4, which is a core molecule mediating the intracellular TGF-β/SMAD signaling transduction pathway, led to the inhibition of the proliferation of ovarian granulosa cells and the synthesis of estradiol [34]. TGF-β1 exerted its effect on progesterone synthesis by downregulating the expression of key genes involved in the progesterone-producing pathway. Specifically, it decreased the expression of StAR, CYP11A1, and HSD3B1, which in turn led to a lower rate of progesterone synthesis [35,36]. In males, TGF-β reduced the concentrations of progesterone (P4) and prostaglandin (PG) E2 in the culture medium and suppressed expression levels of StAR, CYP11A1, cPGES, and mPGES1 [37]. Enhanced TGF-β-induced SMAD2 activation led to a significant decrease in the mRNA levels of LC-specific genes *Cyp17a1*, *Cyp11a1*, *Nr5a1* (*Sf-1*), and *Insl3*. Conversely, inhibition of the TGF-β pathway blocked the downregulation of gene expression such as Cyp17a1 [38]. Furthermore, KEGG pathway enrichment demonstrated that the TGF-β signaling pathway was suppressed by LbGp treatment. In order to investigate whether LbGp upregulates the expression of steroidogenic genes by inhibiting TGF-β, we treated LCs with TGF-β receptor inhibitor SB431542 and found that blocking the TGF-β pathway increased the protein expression of CYP11A1, HSD3B1, HSD17B3, StAR, and SF-1. Hence, our study demonstrated that LbGp increases the synthesis of testosterone in LCs by suppressing the TGF-β signaling pathway.

We also observed an interesting phenomenon, which is that the glycolysis and oxidative phosphorylation processes in LCs were activated by LbGp. This phenomenon has never been reported. Glycolysis and oxidative phosphorylation are the main ways of glucose metabolism and ATP synthesis, providing energy for cell proliferation and migration [39,40]. Therefore, the proliferation of LCs driven by LbGp was attributed to an enhancement in glucose metabolism. Whether there is an association between an enhancement in glucose metabolism and an increase in testosterone synthesis under LbGp treatment requires further investigation. In cancer, phosphofructokinase 1 (PFK1), the enzyme that controls the initial step of glycolysis, attaches to the transcription factors YAP/TAZ and boosts their transcriptional activity [41]. Therefore, more in-depth research is needed in the future to determine whether glucose metabolism regulates testosterone synthesis.

## 5. Conclusions

**Figure 7:**
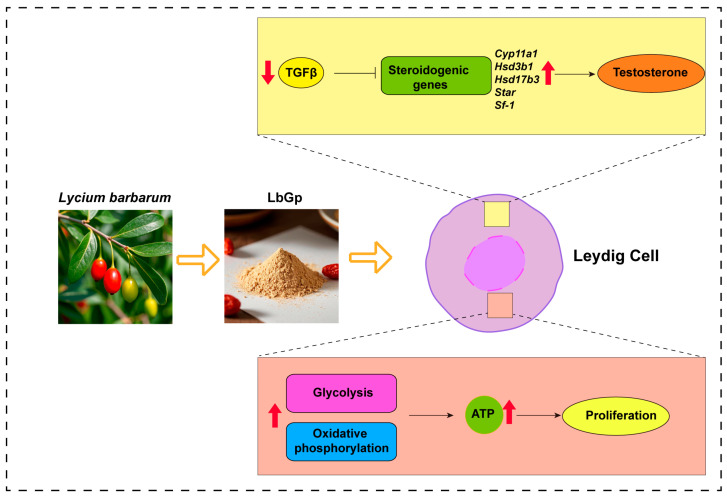
Schematic diagram of the mechanism by which LbGp promotes testosterone synthesis and proliferation in Leydig cells.

In summary, our study identified that LbGp increased the mRNA and protein levels of steroidogenic genes (*Cyp11a1*, *Hsd3b1*, *Hsd17b3*, *Star*, and *Sf-1*) and promoted the synthesis of testosterone by down-regulating the TGF-β signaling pathway. In addition, it promoted cell proliferation by enhancing glucose metabolism (Figure 7). Our research placed significant emphasis on the fact that LbGp holds substantial potential in the treatment of testosterone deficiency.

## Data Availability

The data presented in this study are available on request from the corresponding author.

## Supplementary Materials

The following supporting information can be downloaded at: https://www.mdpi.com/article/10.3390/biom15030425/s1, Figure S1: Steroids detected by UHPLC-MS/MS; Figure S2: LbGp Promotes the Expression of Steroidogenic proteins; Figure S3: SB4 increases the expressions of steroidogenic proteins; Table S1: Antibodies; Table S2: Primers for qRT-PCR; Table S3: Steroids detected in Leydig cells. Supplementary File S1: Original Western blot images.

## Abbreviations

The following abbreviations are used in this manuscript:

LbGp	*Lycium barbarum* glycopeptide
LCs	Leydig cells
RNA seq	RNA sequencing
ATP	Adenosine 5′-triphosphate
TD	Testosterone deficiency
LBP	*Lycium barbarum* polysaccharide
FBS	Fetal bovine serum
TBST	Tris buffered saline with Tween 20
ECL	Enhanced chemiluminescence
qRT-PCR	Quantitative real-time polymerase chain reaction
CCK8	Counting Kit-8
SB4	SB431542
DHT	Dihydrotestosterone
StAR	Steroidogenic acute regulatory
CYP11A1	Cytochrome P450 family 11 subfamily A member 1
HSD3B1	Hydroxy-delta-5-steroid dehydrogenase, 3β-and steroid delta-isomerase 1
HSD17B3	Hydroxysteroid 17β dehydrogenase 3
SF-1	Steroidogenic factor 1
CYP17A1	Cytochrome P450 family 17 subfamily A member 1
LHCGR	Luteinizing hormone receptor
DEGs	Differentially expressed genes
PCA	Principal component analysis
