# Supplementary material for: Lycium barbarum Glycopeptide Promotes Testosterone Synthesis and Glucose Metabolism in Leydig Cells of the Testis
Source: Biomolecules. 2025 Mar 17;15(3):425. doi: 10.3390/biom15030425 (PMC11940756; doi:10.3390/biom15030425)
Supplement: Supplementary file 1 [file biomolecules-15-00425-s001.zip › biomolecules-3494954-supplementary.pdf]

Supplemental Information

Lycium barbarum glycopeptide promotes testosterone synthesis and glucose metabolism in Leydig cells of the testis

Jinlian Liang [1†], Tianchan Peng [2, 3†] , Jinrong Hu[2, 3], Kwok Fai So[4, 5, 6], Hongyi Zhang[7], Guobin Chen[2, 7*], and Yuan-Wei Zhang[1*]

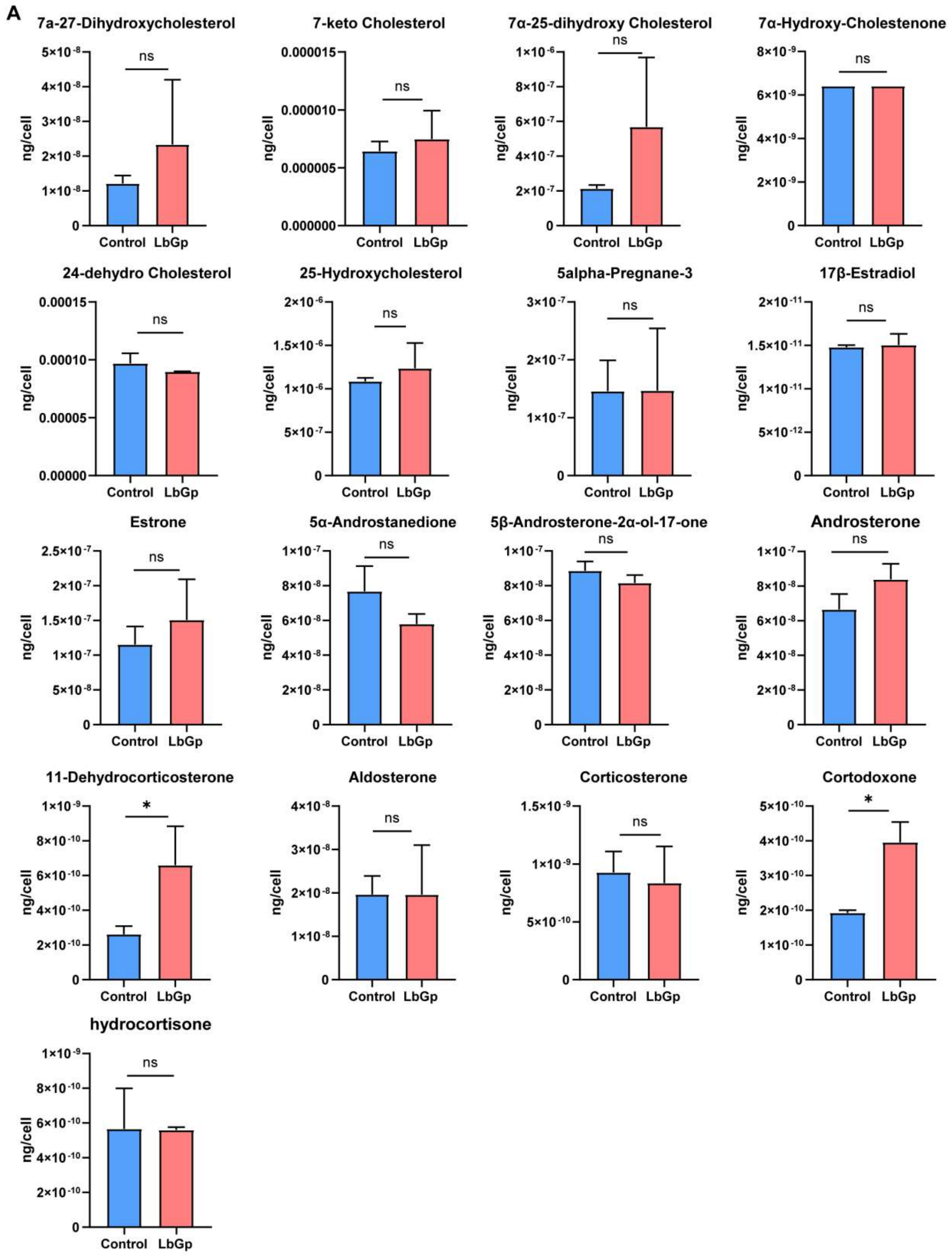

Figure S1 Steroids detected by UHPLC-MS/MS

A. The concentrations of various steroids in LCs treated with or without 100 μg/mL LbGp for 48 hours detected by UHPLC-MS/MS, *$p < 0.05$, "ns" means not significant.

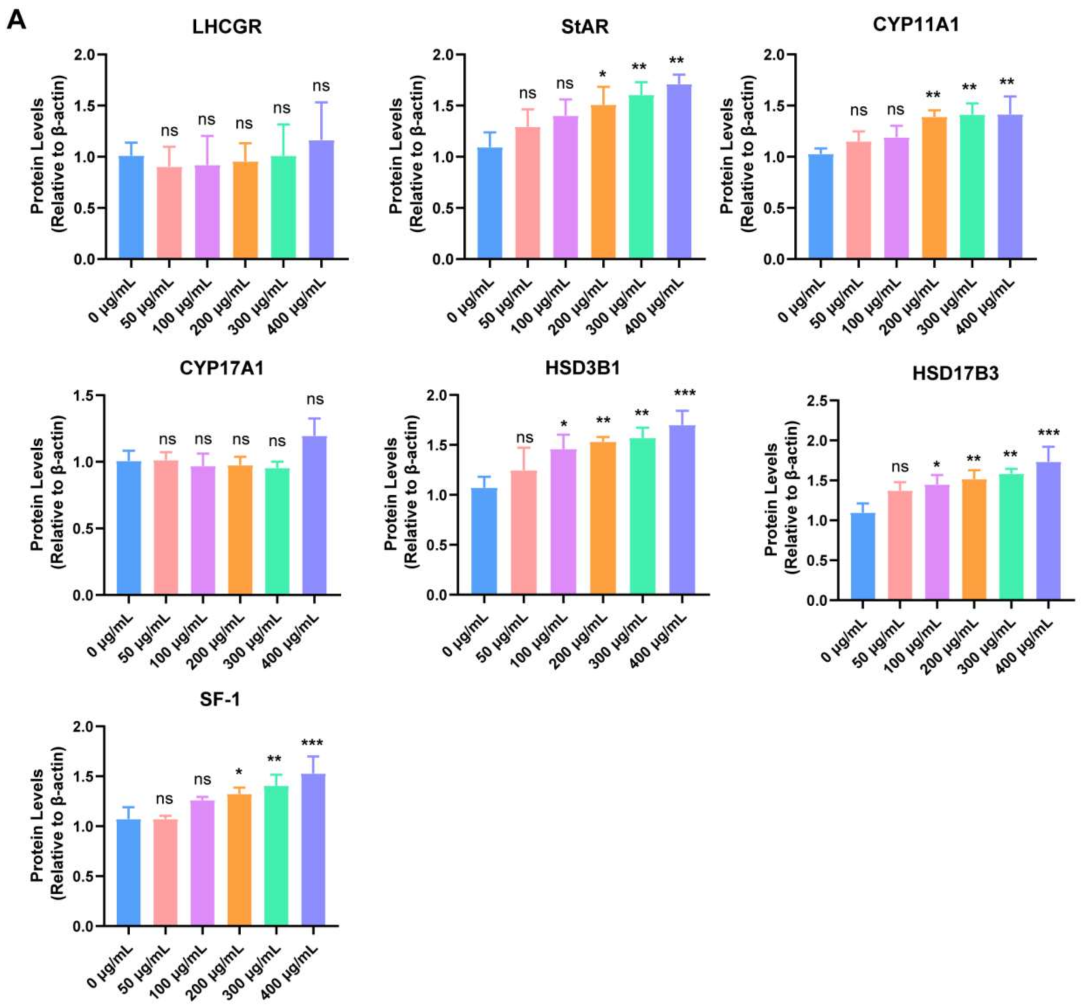

Figure S2 LbGp Promotes the Expression of Steroidogenic proteins

A. The statistic of protein levels of LHCGR, StAR, CYP11A1, CYP17A1, HSD3B1, HSD17B3, and SF-1 in LCs treated with different concentrations of LbGp detected by western blotting in Figure 2B. Data are presented as the mean ± SD from at least three independent experiments. *$p < 0.05$, **$p < 0.01$, ***$p < 0.001$, ****$p < 0.0001$, “ns” means not significant.

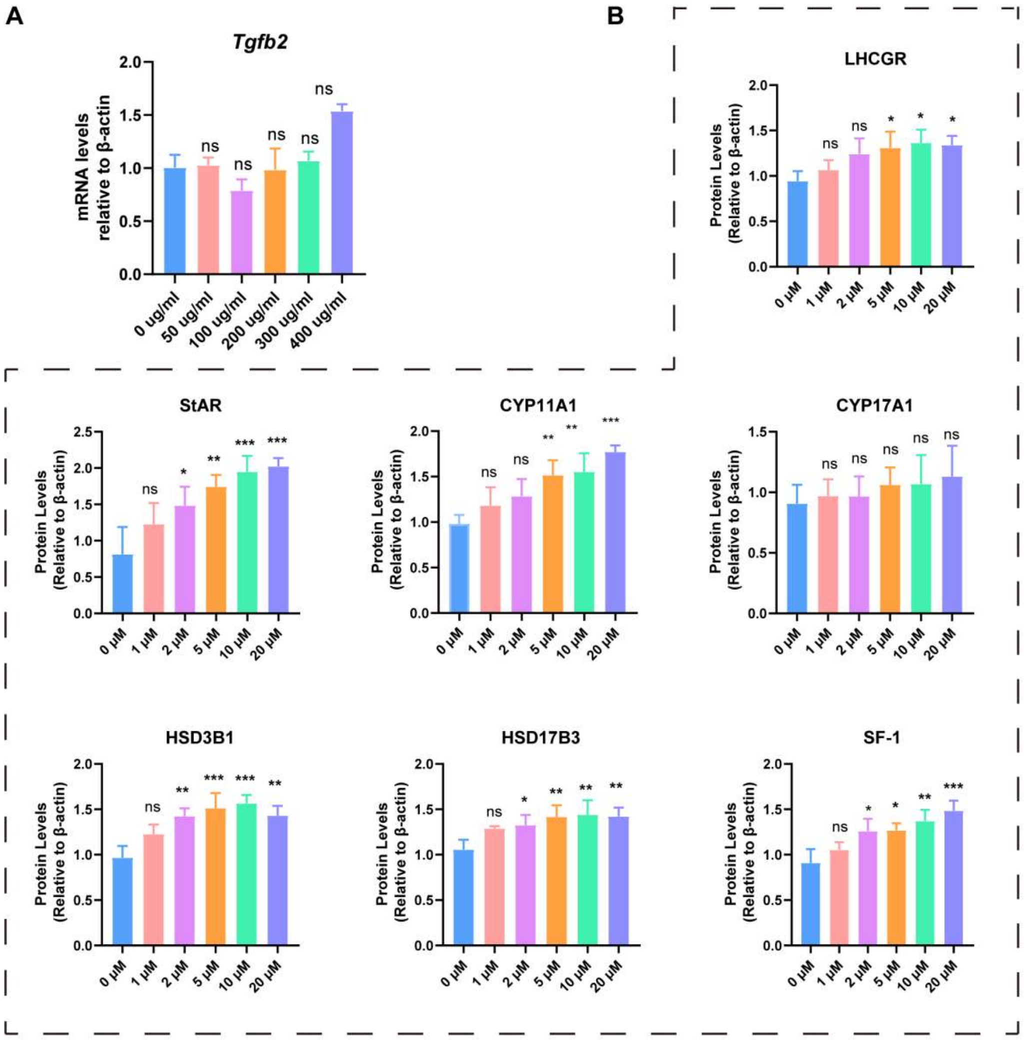

Figure S3 SB4 increases the expressions of steroidogenic proteins

A. The mRNA levels of *Tgfb2* in LCs treated with 0, 50, 100 200, 300 and 400 μg/mL LbGp for 24 hours detected by qRT−PCR in Figure 5A.

B. The statistic of expression of steroidogenic proteins in LCs treated with 0, 1, 2, 5, 10 and 20 μM SB4 for 48 hours detected by western blotting in Figure 5C. Data are presented as the mean ± SD from at least three independent experiments. $*p < 0.05$, $**p < 0.01$, $***p < 0.001$, $****p < 0.0001$,"ns" means not significant.

Table S1 Antibodies

Name	Product code	Manufacturer
LHCGR	19968-1-AP	Proteintech Group
CYP11A1	13363-1-AP	Proteintech Group
CYP17A1	94004T	Cell Signaling Technology
HSD3B1	HY-P81196	MedChemExpress
HSD17B3	ab126228	Abcam
StAR	8449T	Cell Signaling Technology
SF-1	12800T	Cell Signaling Technology
Goat Anti Rabbit IgG(488)	ab150077	Abcam

Table S2 Primers for qRT-PCR

Name	Sence primer (5'-3')	Anti-sence primer
Lhcgr	GATGCACAGTGGCACCTTCCAG	GTGGCGATGAGCGTCTGAATGG
Star	CCCAAAGAAGGCATAGCAAG	GCTGAATCCCCCAAACTTCT
Cyp11a1	CCAGTGTCCCCATGCTCAAC	TGCATGGTCCTTCCAGGTCT
Cyp17a1	AGCACCTAGAGGCCGAATCT	TGTCTCACCCTTCATTGCTG
Hsd17b3	CAAGATGACCAAGACCGCCGATG	GAGCAAGGCAGCCACAGGATTC
Hsd3b1	TGATCTTTTCAGCCACCACCA	AGCCGCTCAGTTCAGAATGT
Sf-1	TAGCCACTGCCCTACCTGAG	AAGAAGCCCTTGCAGCTCTC
Tgfb3	GGAAATGGGTCCACGAACCT	CCTCTGGGTTCAGGGTGTTG
Bmp4	GAGCCATTCCGTAGTGCCAT	ACGACCATCAGCATTCGGTT
Bmp6	CTCTATCGGCGGCTCAAGAC	TTCGTCGTCATTGGACAGGG
Hk2	ACCCGGAGTTGTTCTGCTTT	GGTCTAGCTGCTTAGCGTCC
Pfkl	TGAGGATGGCTGGGAGAACT	TGAACCACCAGATCCTTCACG
Aldoa	GCTCCTTAGTCCTTTCGCCTAC	AACTCTGTCTGTTGCTGGGTG
Tpi1	TCGGGGAGAAGCTAGACGAA	TGAGCCACCCCATCATTGAC
Pkm	GTATCGCAGCAGGAACCGAA	CTCCACAGATCGGGAAGCAG

Ldha	CGGTTCCGTTACCTGATGGG	TTCCACTGCTCCTTGTCTGC
Atp5mc3	GGCCAGAGACTAGGACTGGA	ATACCAGCACCAGAACCAGC
Ndufa4l2	AAAAGACACCCTGGGCTCATC	CATTGGGACTCAGGCGGTT
Ndufv3	CGGCACTGTCCAAAAACTCAC	TGGGGTGGAGGCATAACTTTC
Sdhd	ACCTTTGCTGGGCTTTGCTA	GGGCTTATCCTTCATCCCCC
Uqcrfs1	TGCTTCTGCTGACGTACTGG	TATGGCGCACAAACAGAGGT
β-actin	GAGCGCAAGTACTCTGTGTG	AACGCAGCTCAGTAACAGTC

Table S3 Steroids detected in Leydig cells

NO.	Steroids	Detected	NO.	Steroids	Detected
1	Testosterone	√	20	Progesterone	√
2	DHT	√	21	17a-Hydroxyprogesterone	×
3	Androsterone	√	22	7a-Hydroxy-Cholestenone	√
4	5β-Androsterone-2α-Ol-17-one	√	23	7-keto Cholesterol	√
5	DHEA	√	24	7a,27-Dihydroxycholesterol	√
6	Androstenedione	√	25	11-Oxo Etiocholanolone	×
7	5α-Androstanedione	√	26	24-Hydroxycholesterol	×
8	hydrocortisone	√	27	7a,25-dihydroxy Cholesterol	√
9	11-Dehydrocorticosterone	√	28	Cholesterol	√
10	Deoxycorticosterone	×	29	25-Hydroxycholesterol	√
11	Corticosterone	√	30	24-dehydro Cholesterol	√
12	Cortodoxone	√	31	Lathostero	×
13	Aldosterone	√	32	20(S)-Hydroxycholesterol	×
14	Cortisone	×	33	Pregnanediol	×
15	Estrone	√	34	Pregnenolone	√
16	2-Methoxyestrone	×	35	17β-Estradiol	√
17	4-Methoxyestrone	×	36	17a-Estradiol	×
18	Pregnenolone	√	37	17a-Ethynyl estradiol	×
19	5alpha-Pregnane-3,20-dione	√	38	16a-hydroxy Estrone	×
