# Supplementary figures and images for: Lycium barbarum Glycopeptide Promotes Testosterone Synthesis and Glucose Metabolism in Leydig Cells of the Testis
Source: Biomolecules. 2025 Mar 17;15(3):425. doi: 10.3390/biom15030425 (PMC11940756; doi:10.3390/biom15030425)

Fig 2B

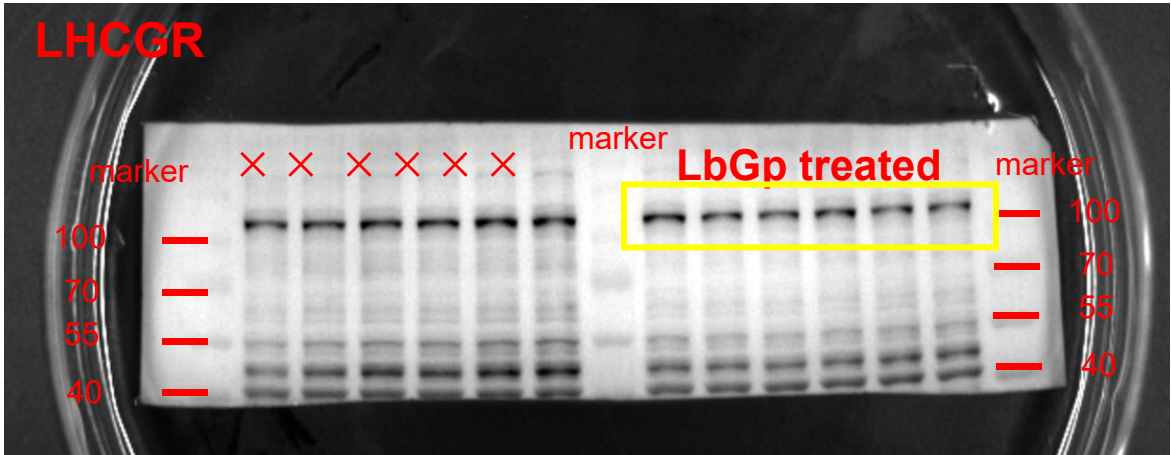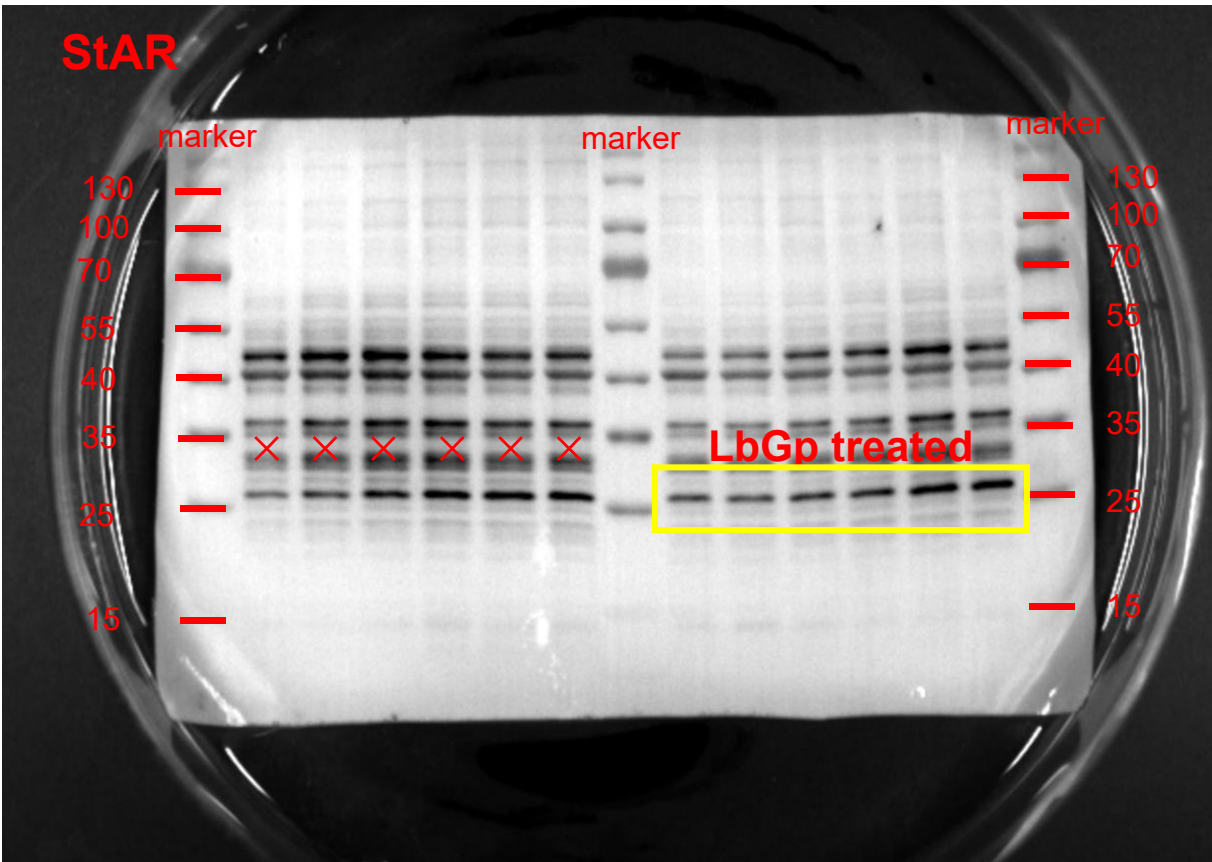

Fig 2B

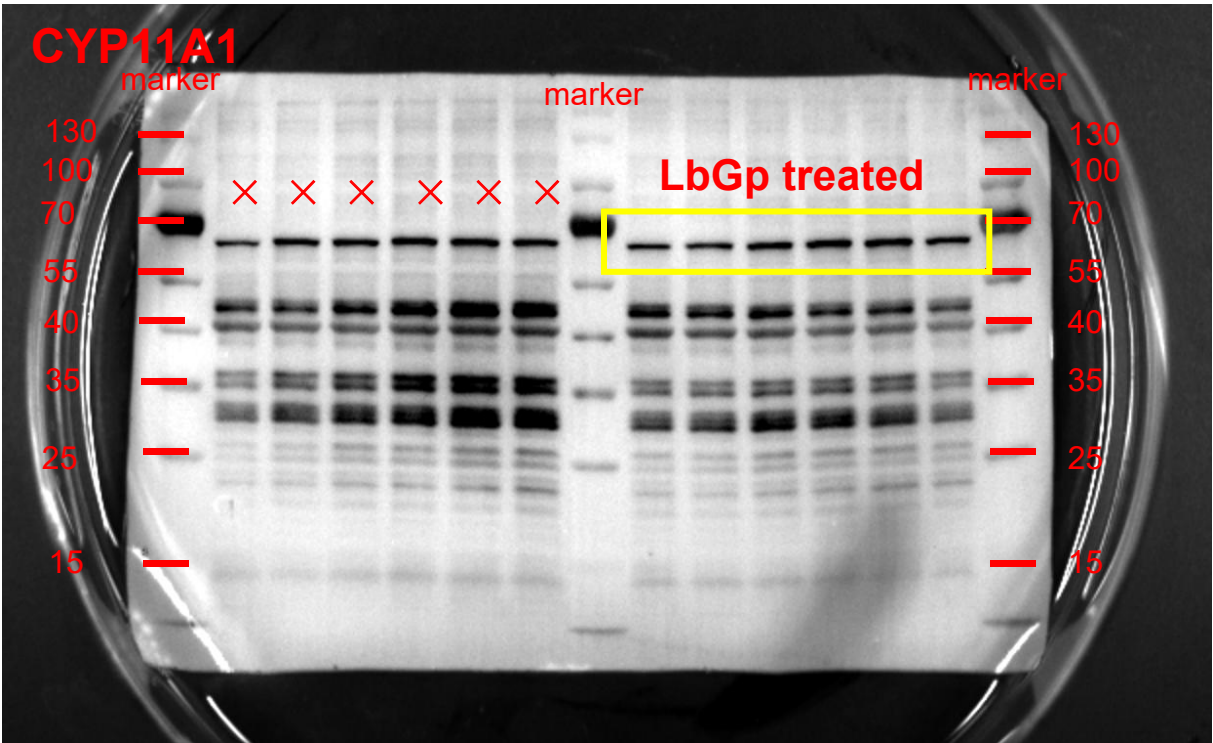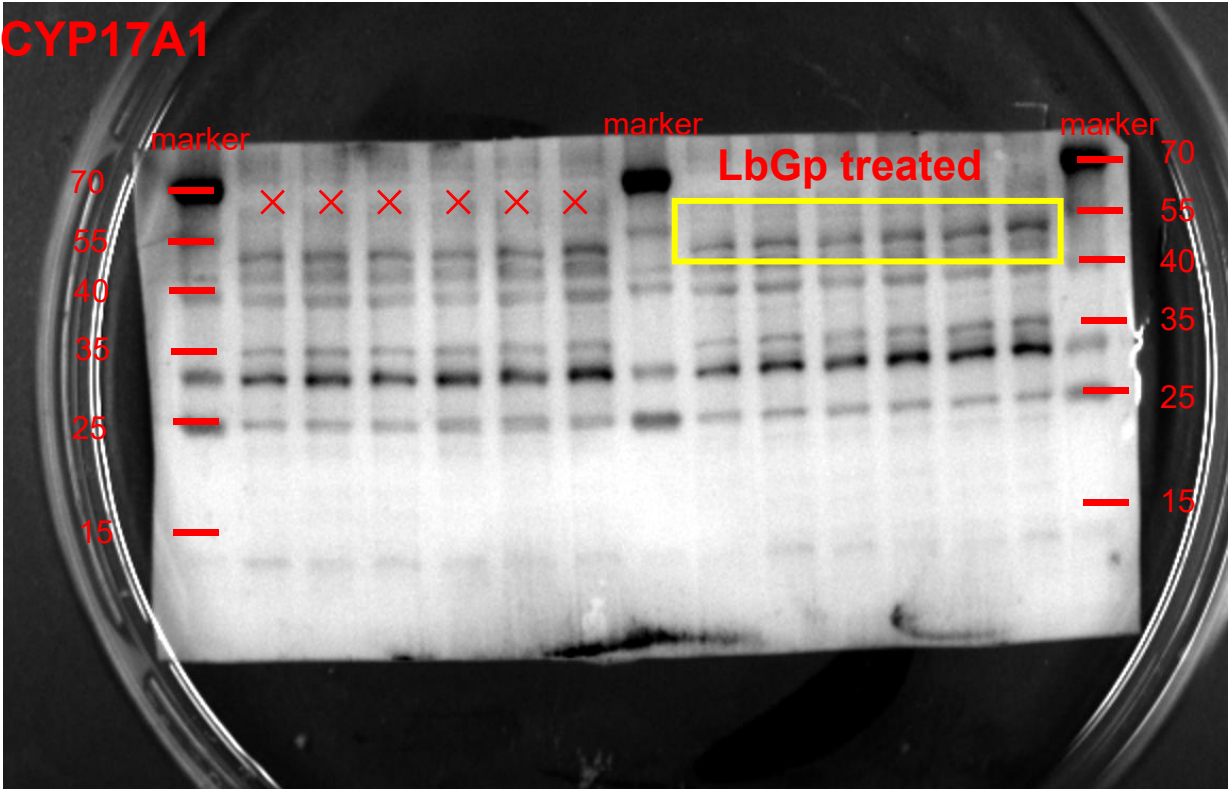

Fig 2B

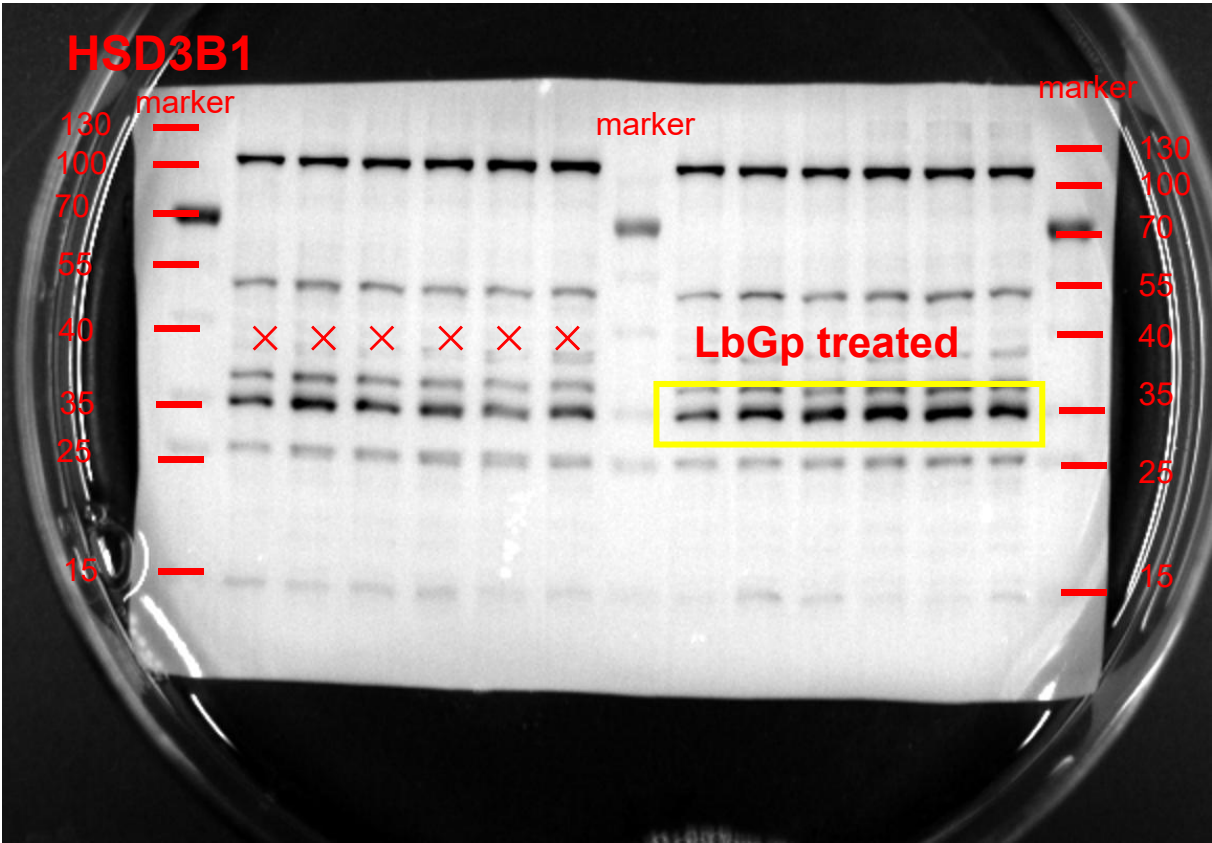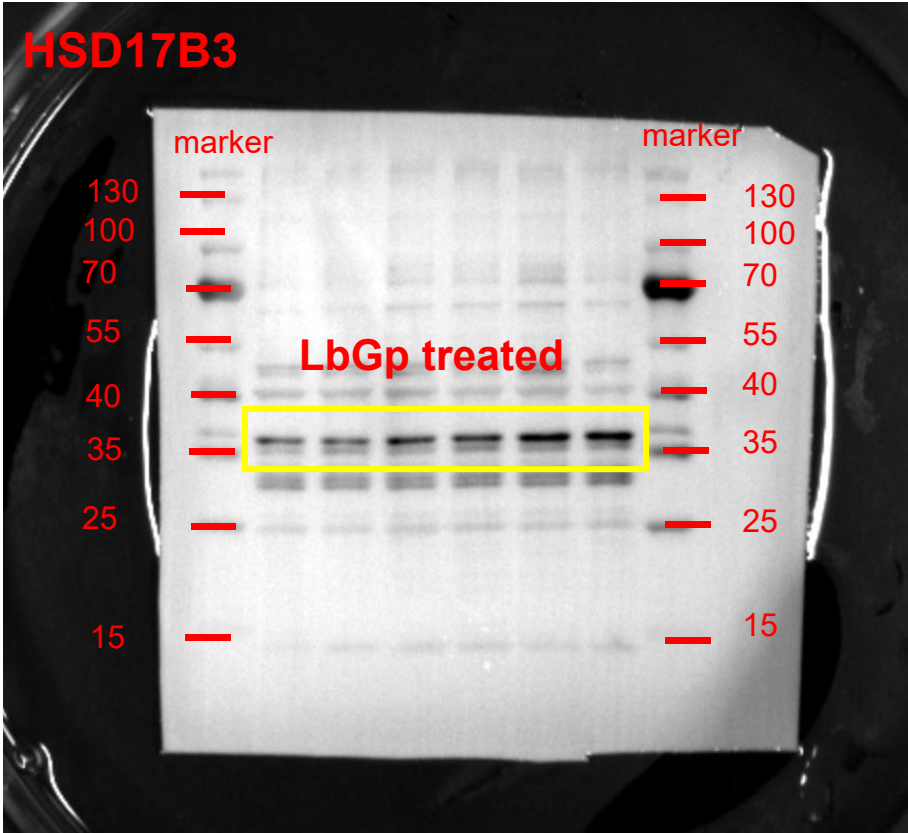

Fig 2B

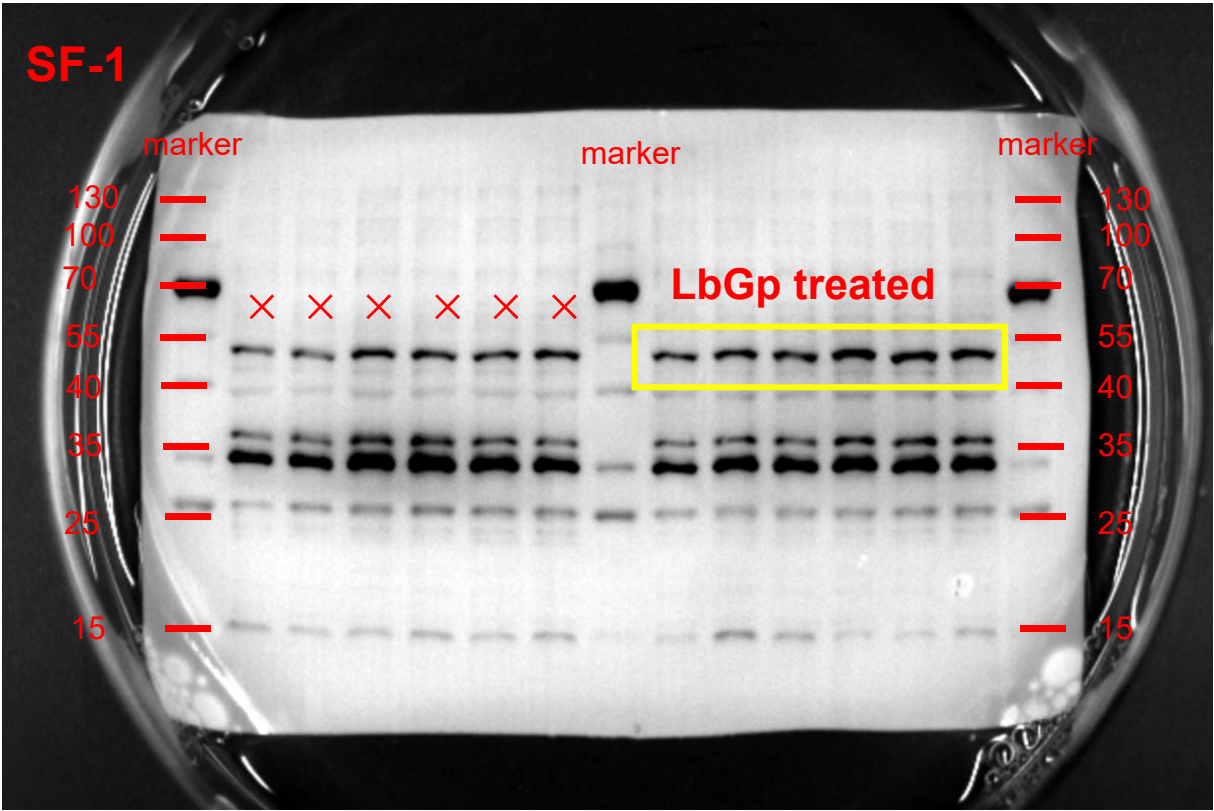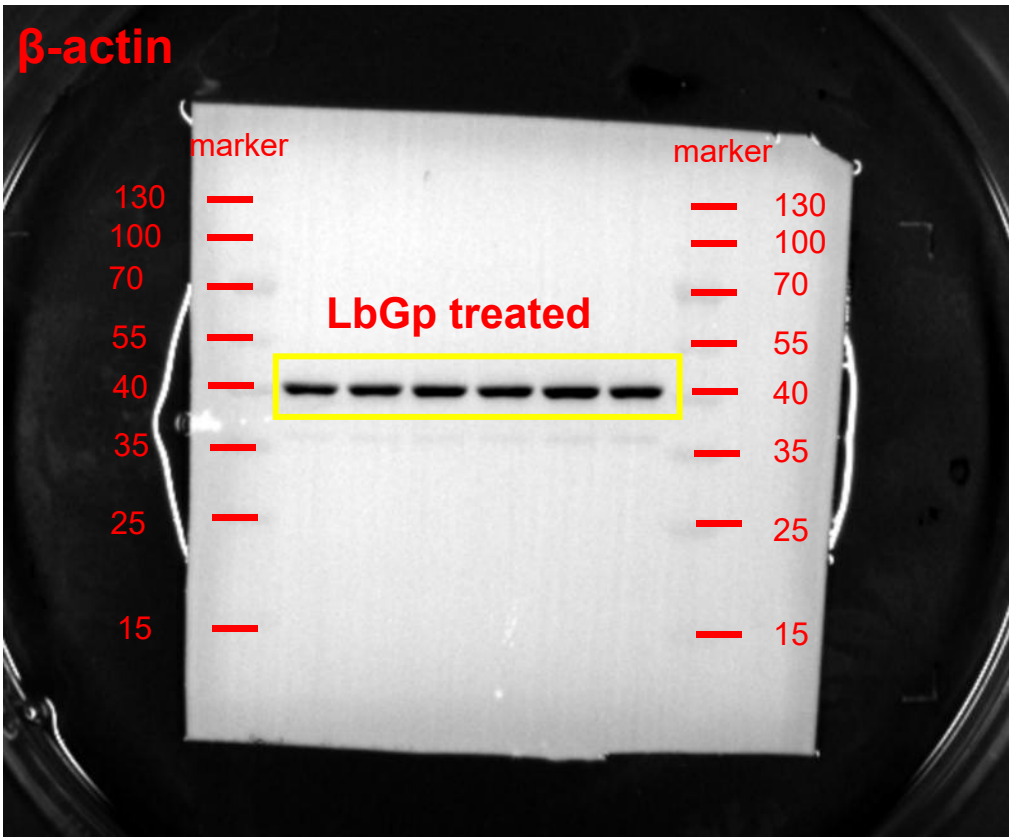

Fig 5C

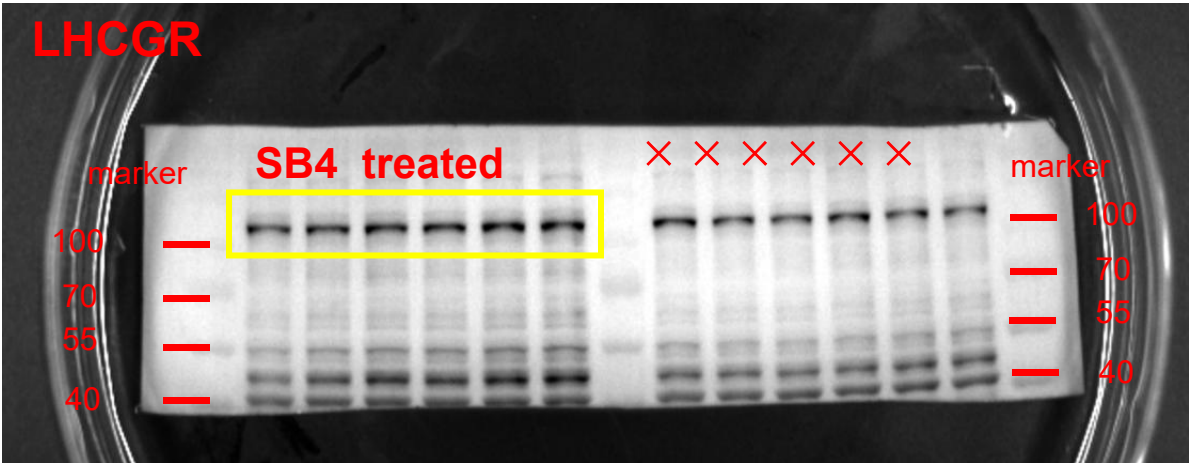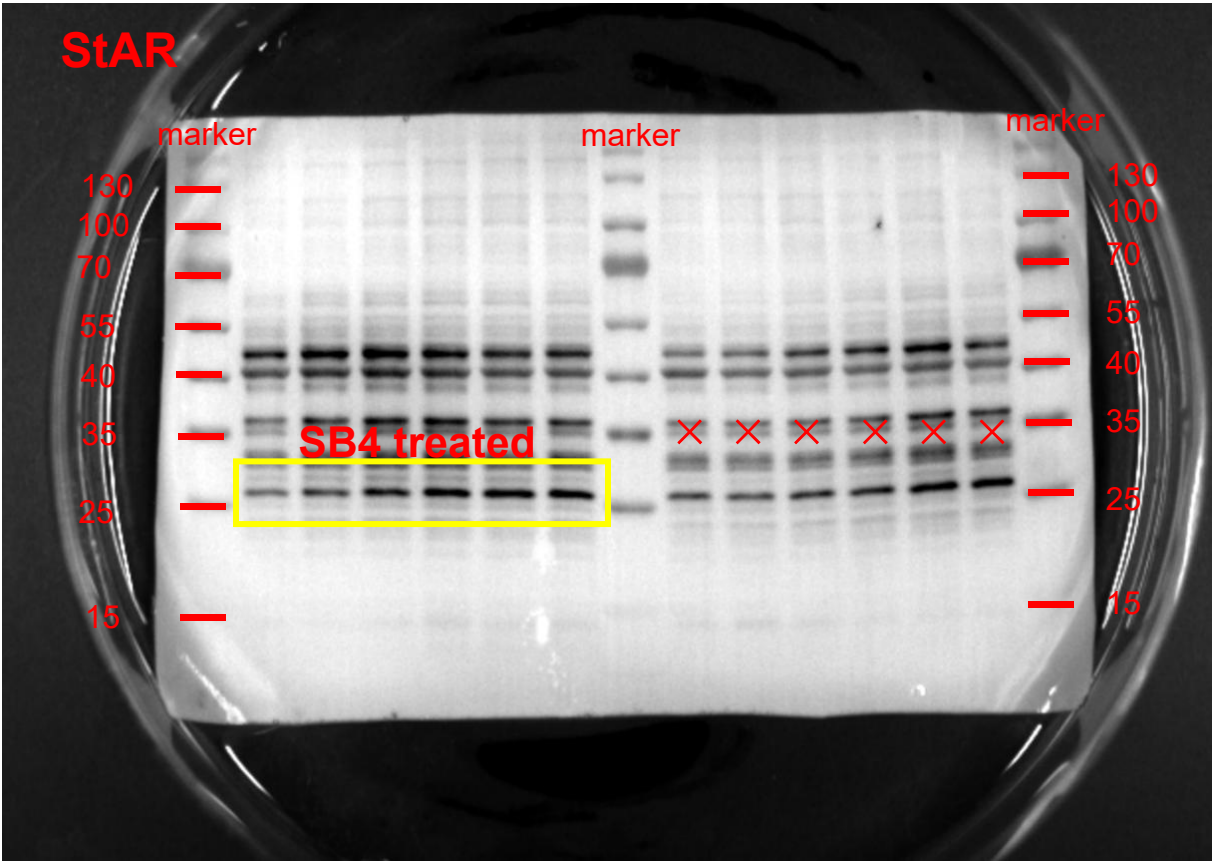

Fig 5C

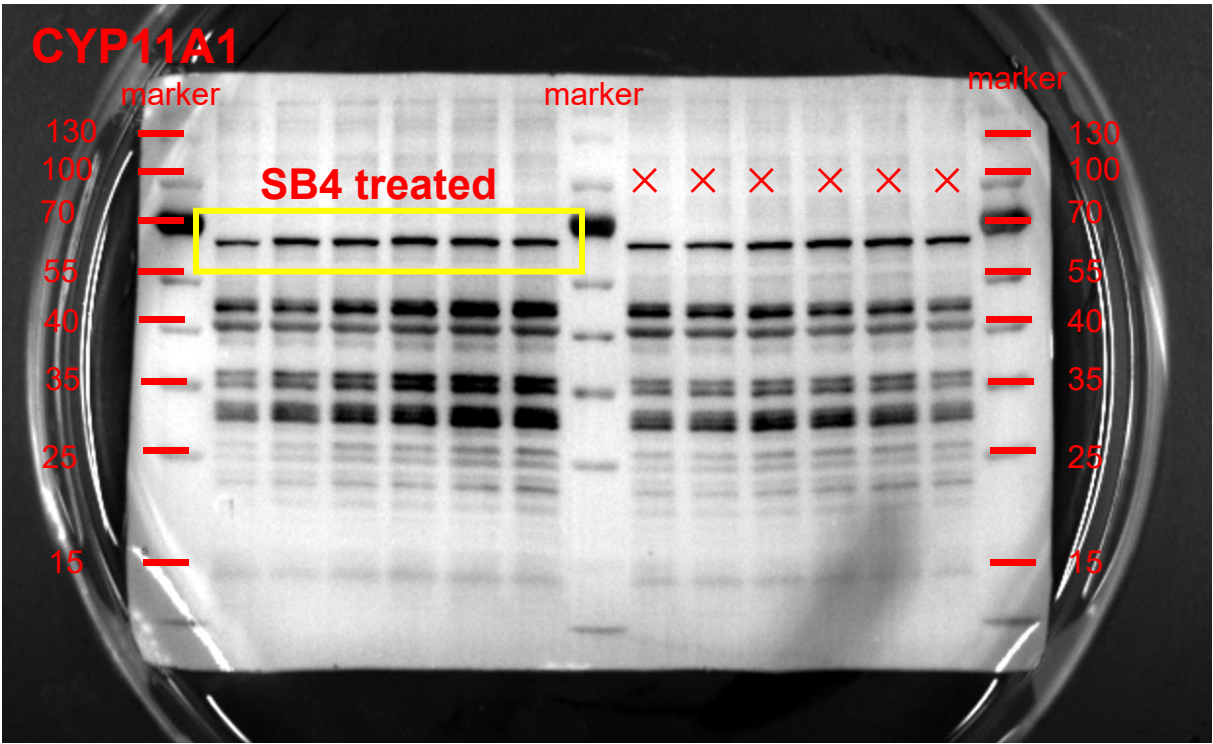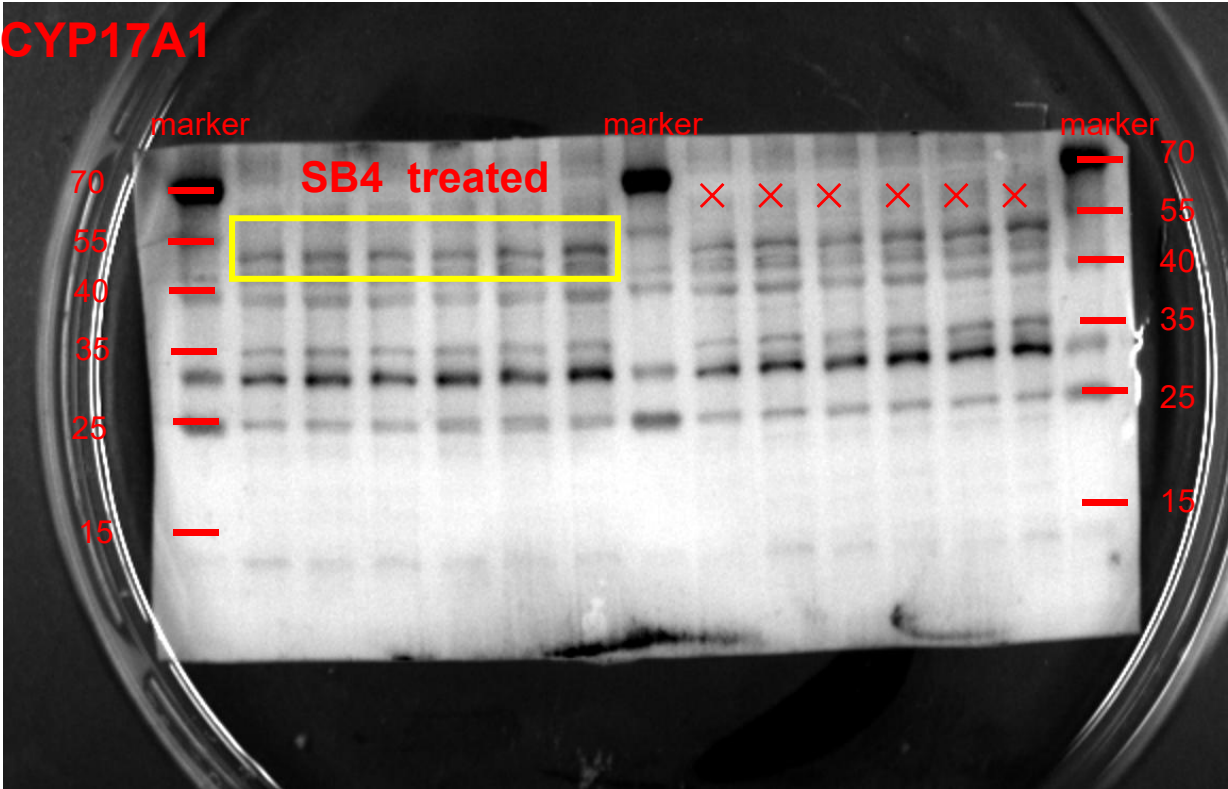

Fig 5C

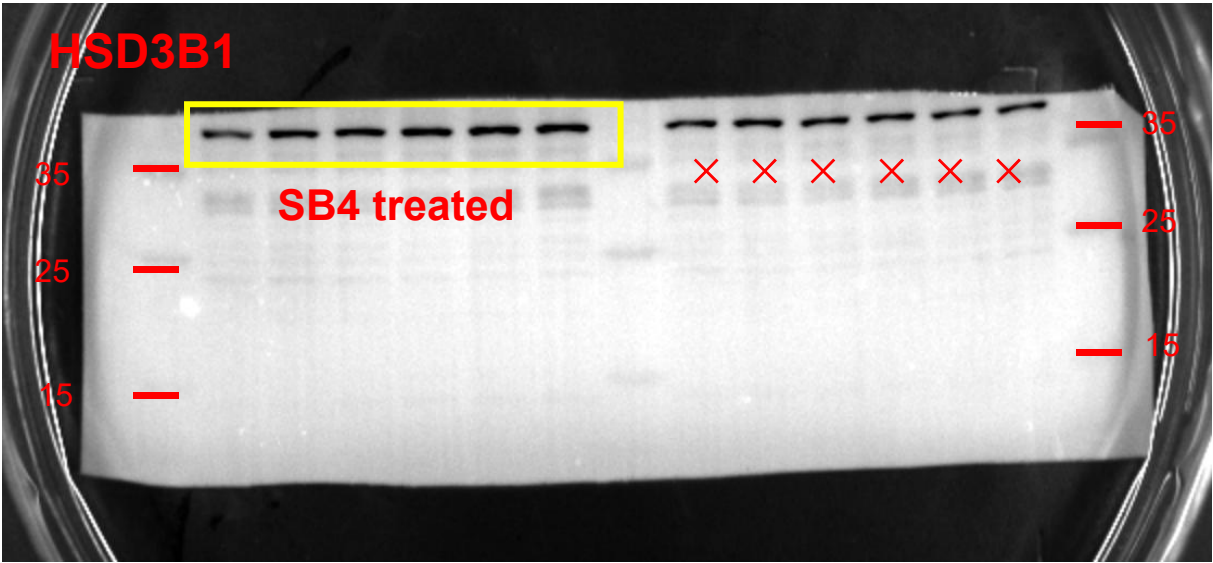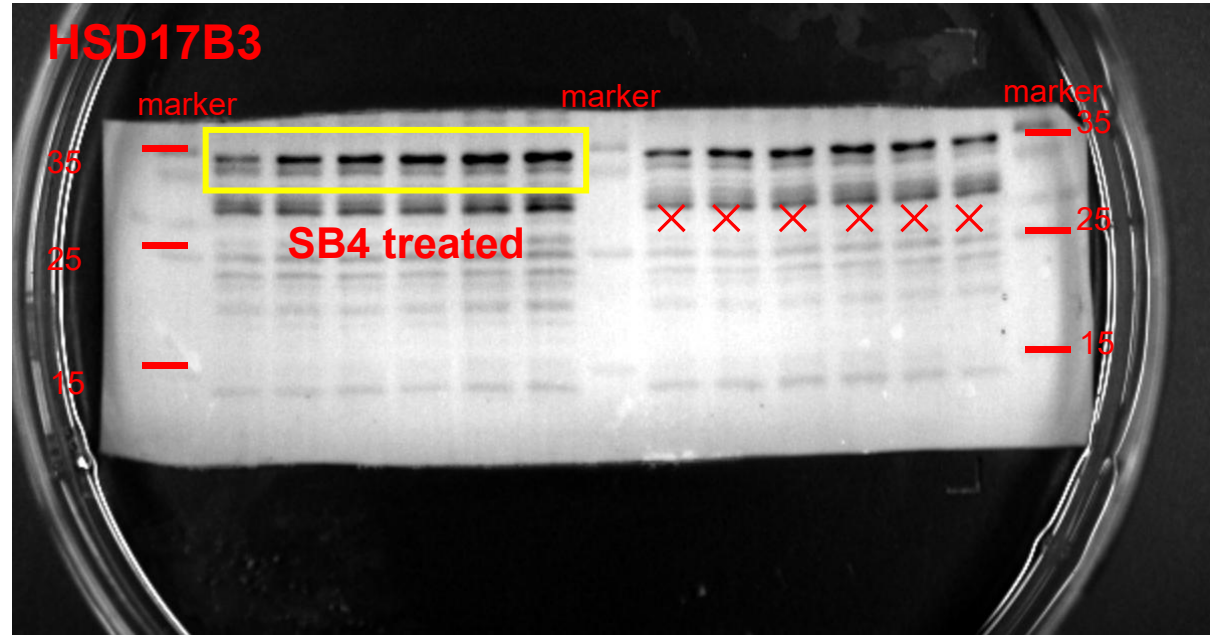

Fig 5C

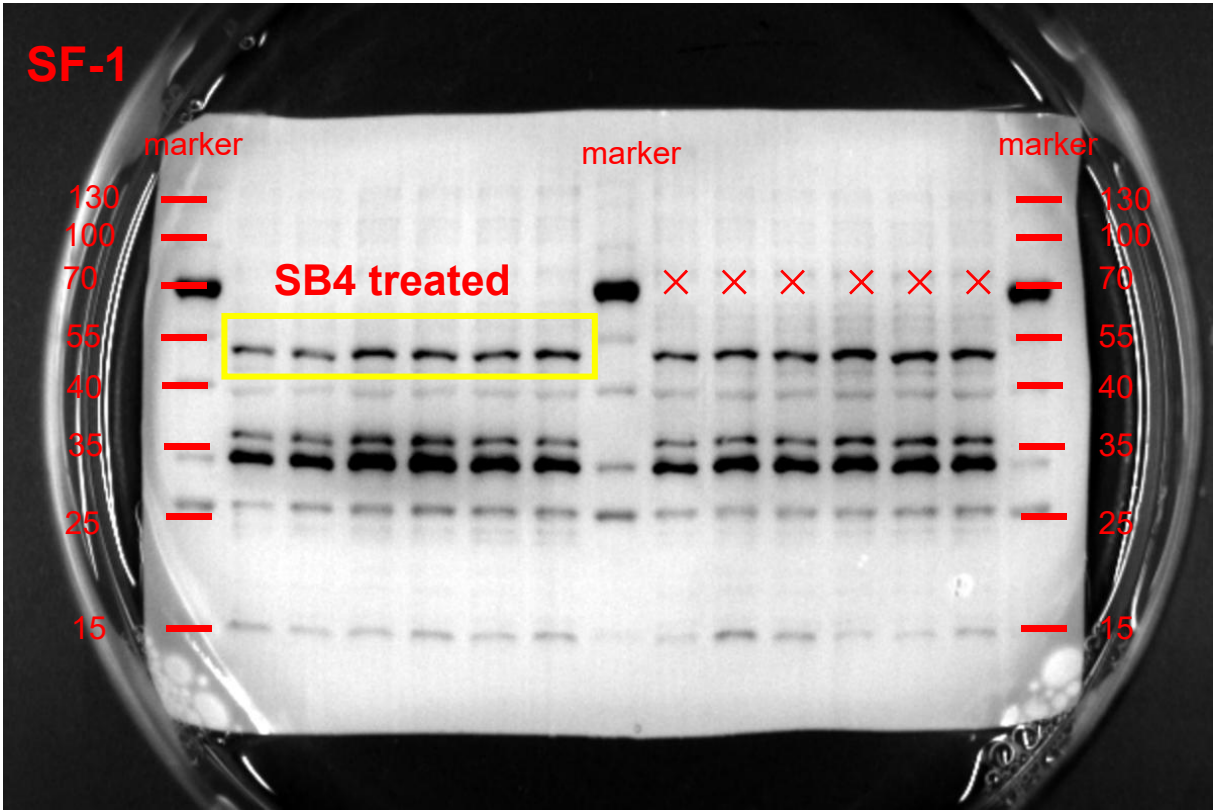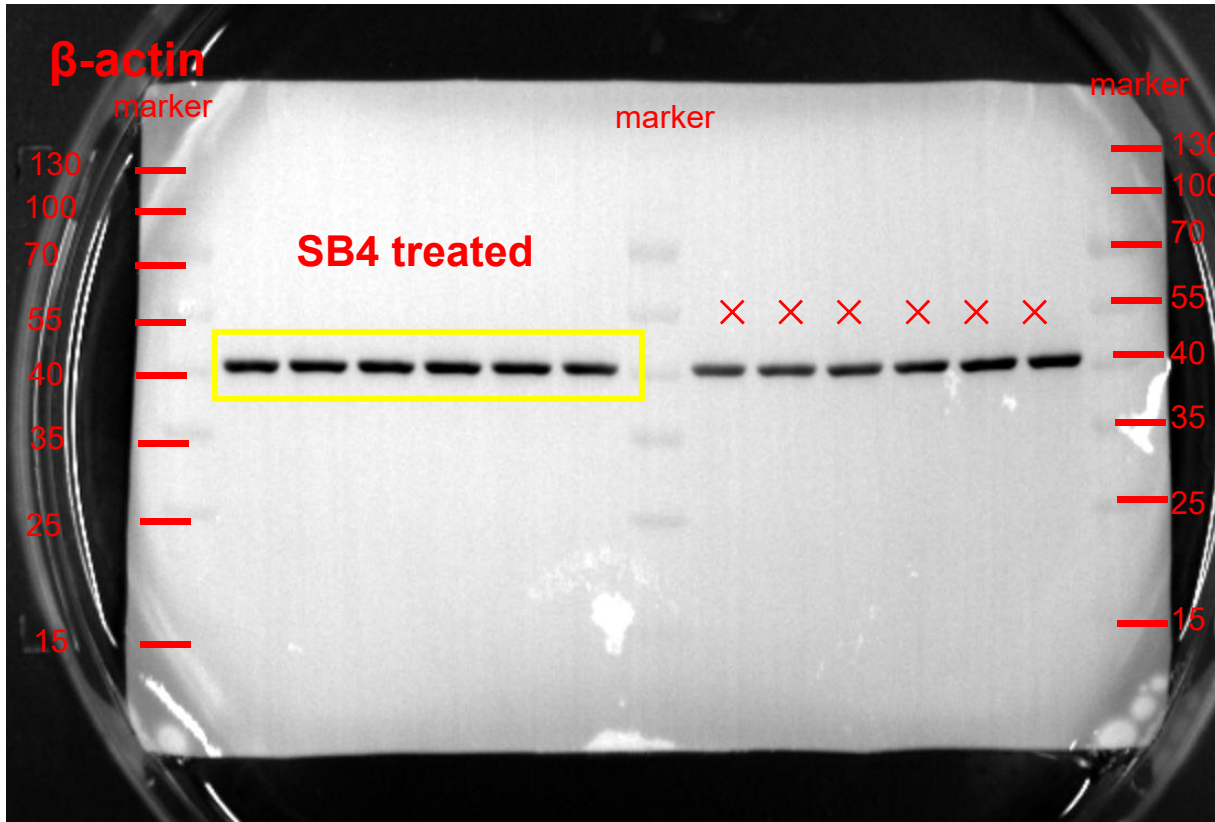

Fig 6C

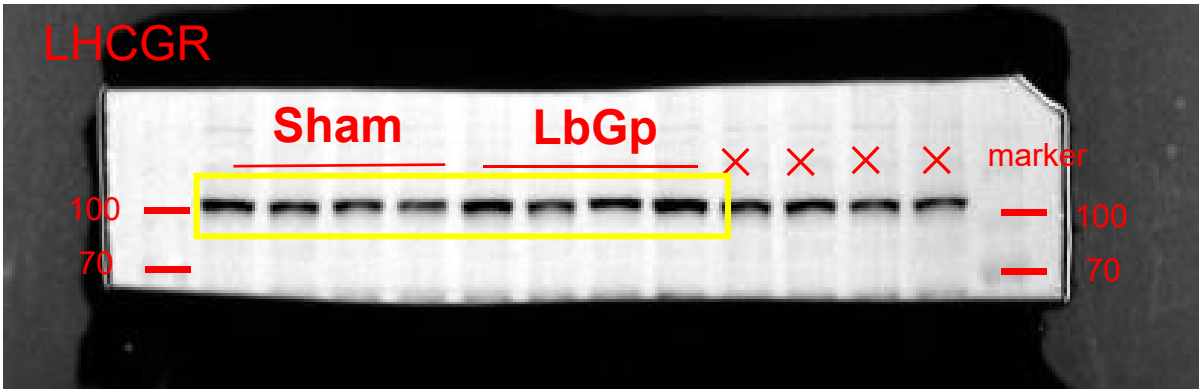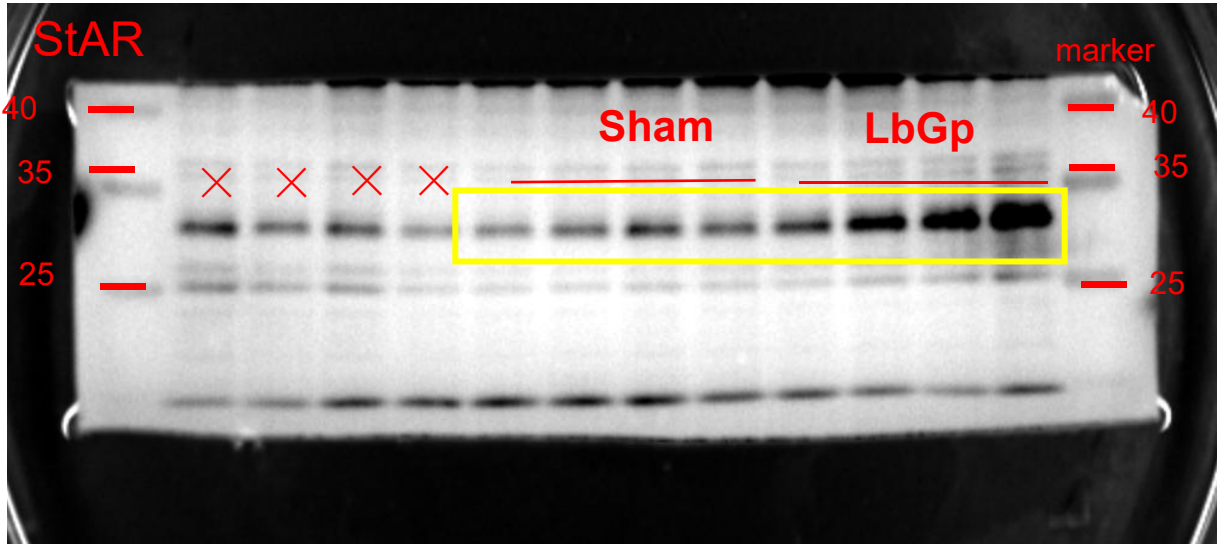

Fig 6C

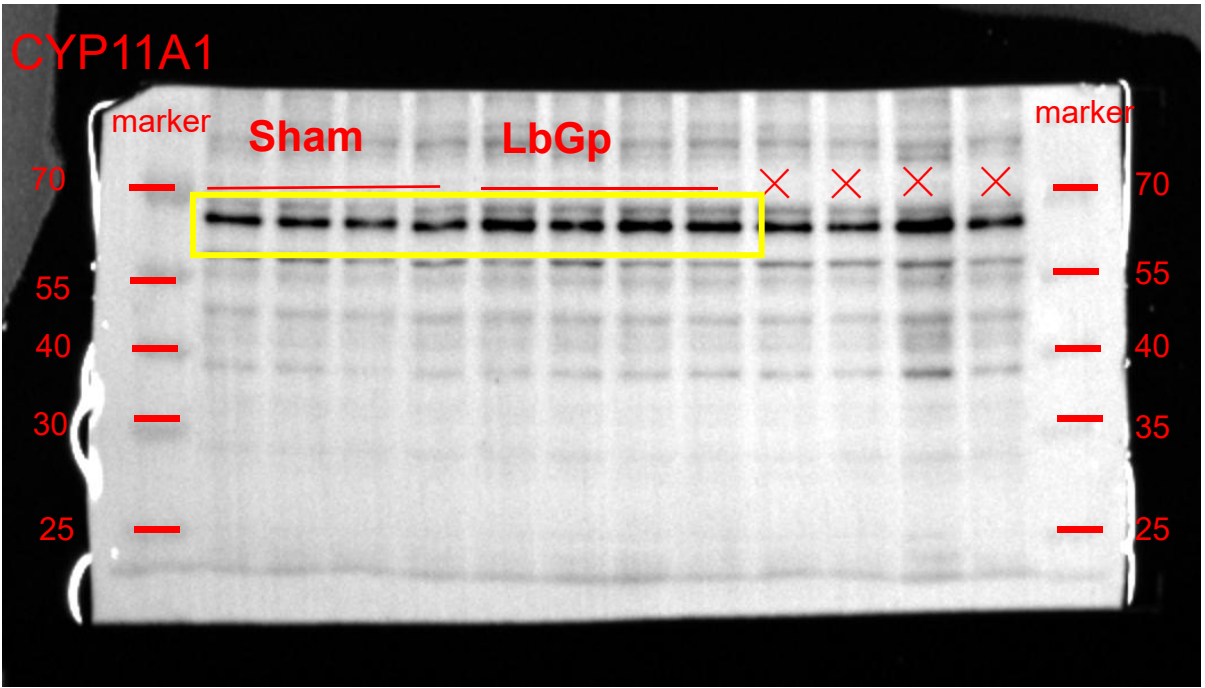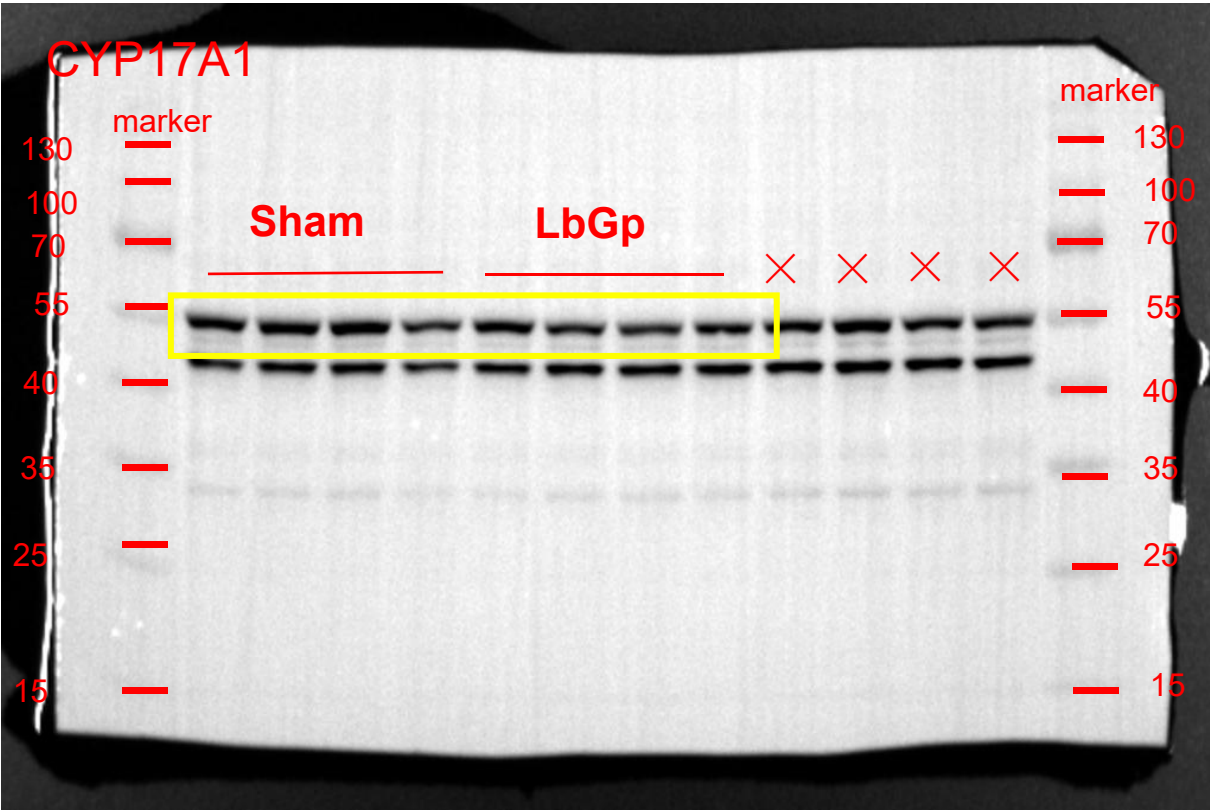

Fig 6C

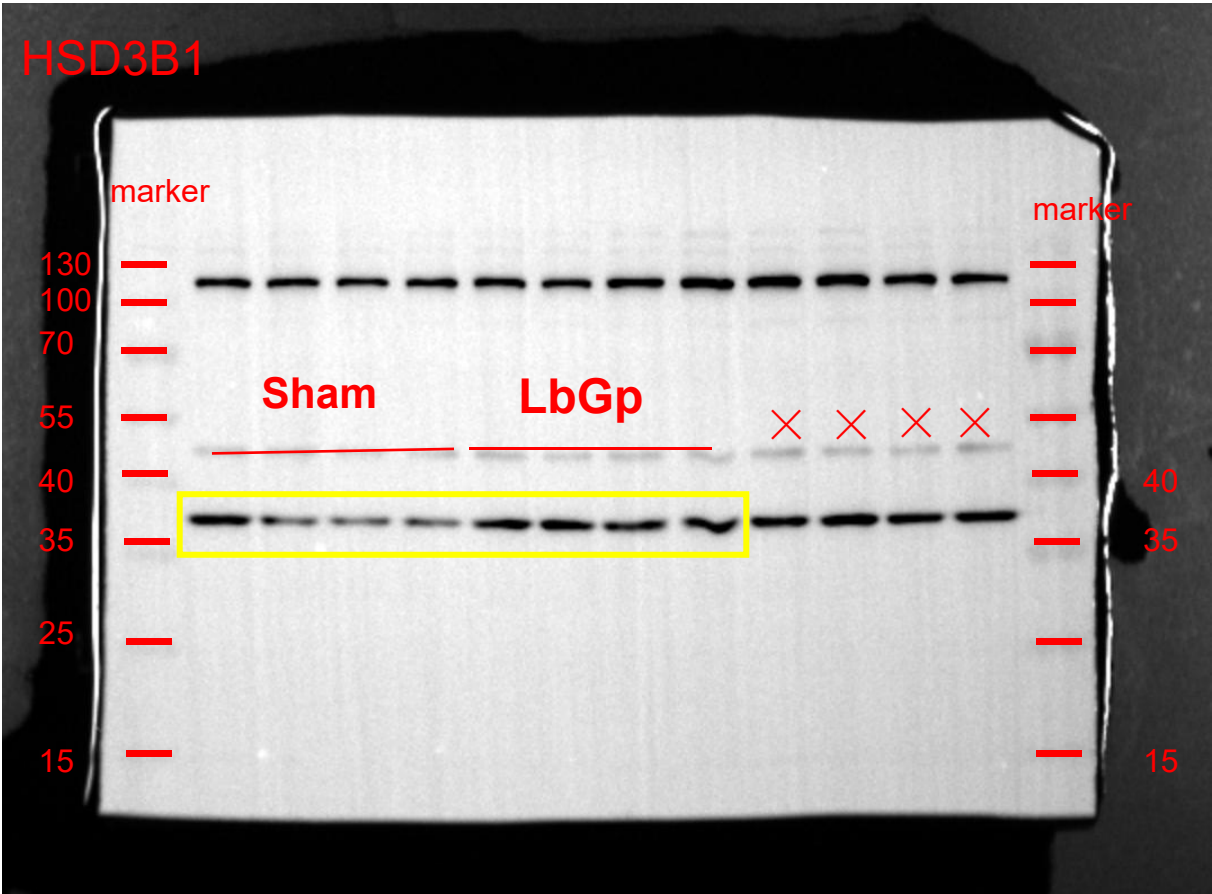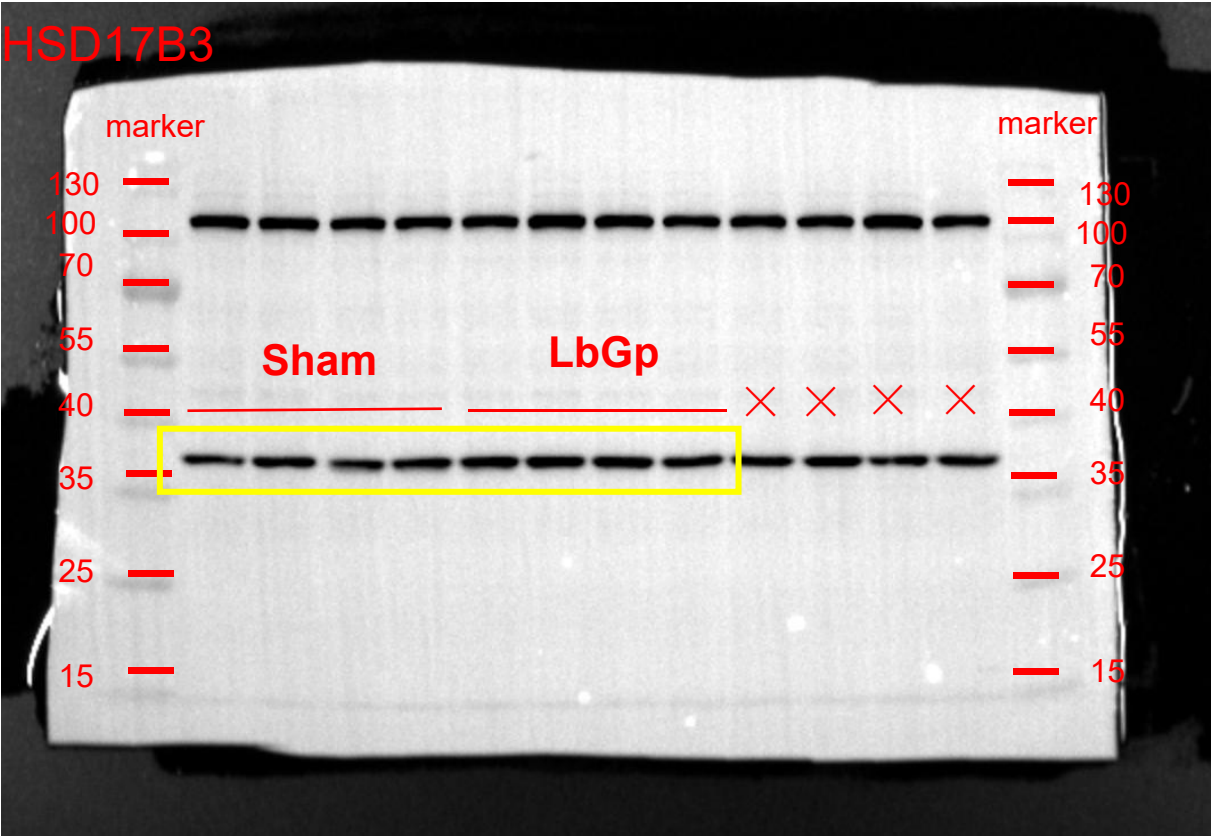

Fig 6C

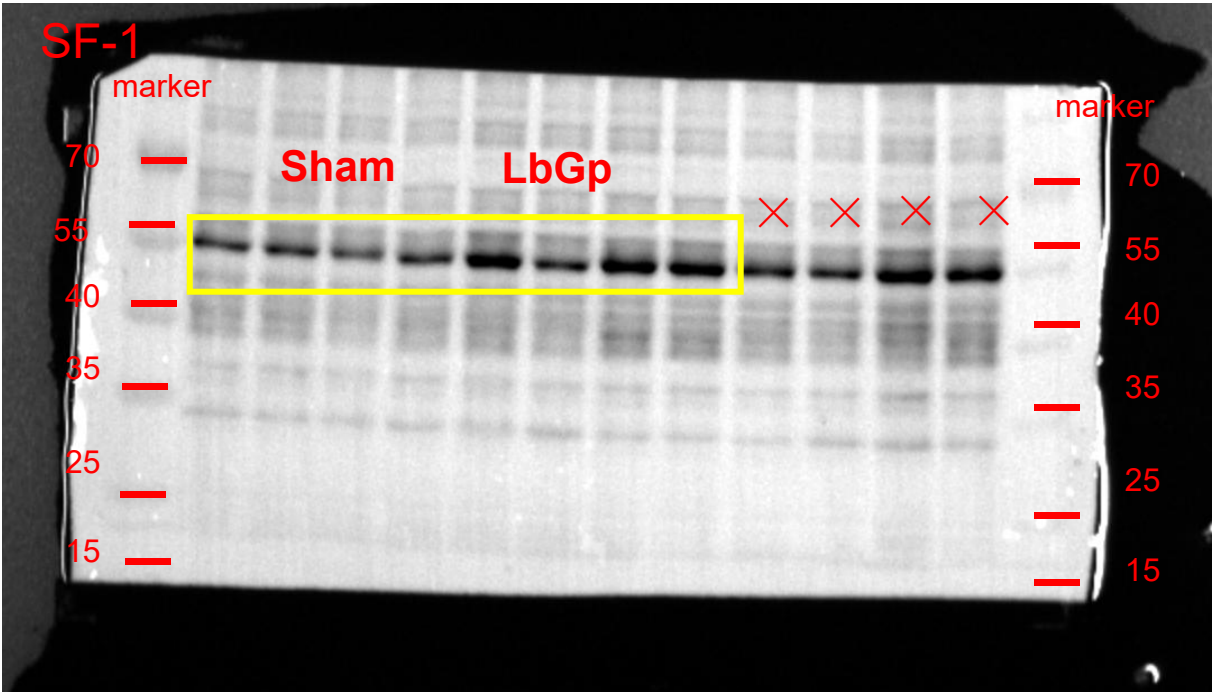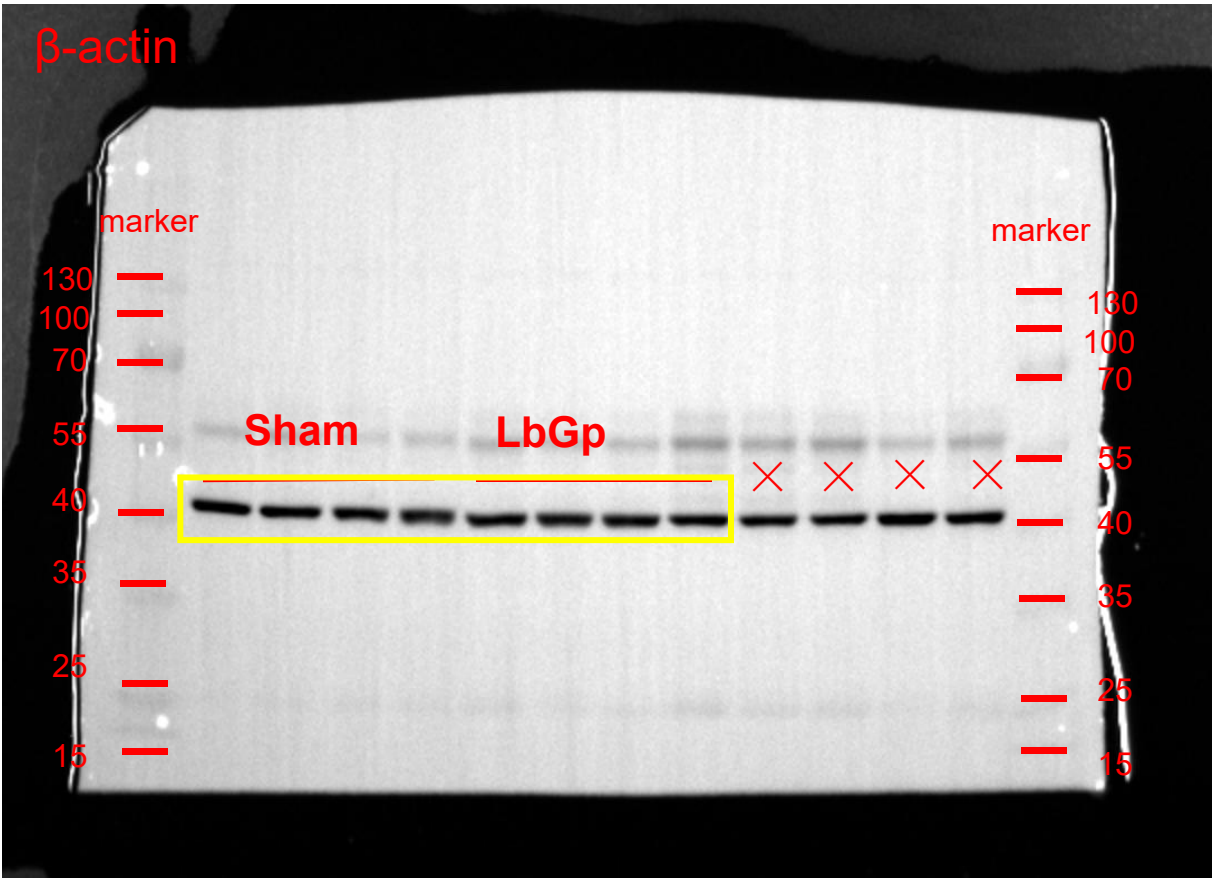

Supplement: Supplementary file 1 [file biomolecules-15-00425-s001.zip › Supplementary File 1-Original Western Blots.pdf]
